# Necroptosis-related genes are associated with prognostic features of kidney renal clear cell carcinoma

**DOI:** 10.1007/s12672-023-00794-0

**Published:** 2023-10-25

**Authors:** Yiduo Wang, Ke-Hao Pan, Ming Chen

**Affiliations:** 1 Affiliated Zhongda Hospital of Southeast University, Southeast University, 87 Dingjia Bridge Hunan Road, Nanjing, China; 2 State Key Laboratory of Molecular Oncology, National Cancer Center/National Clinical Research Center for Cancer/Cancer Hospital, Chinese Academy of Medical Sciences and Peking Union Medical College, Beijing, China; 3 Department of Urology, National Cancer Center/National Clinical Research Center for Cancer/Cancer Hospital, Chinese Academy of Medical Sciences and Peking Union Medical College, Beijing, China; 4 Department of Urology, Lishui District People’s Hospital, Affiliated Zhongda Hospital of Southeast University, 87 Dingjia Bridge Hunan Road, Nanjing, China

**Keywords:** Necroptosis, Kidney renal clear cell carcinoma, Prognosis, Biomarkers

## Abstract

**Introduction:**

Renal clear cell carcinoma is a common type of cancer in the adult urological system. It has a high mortality rate, with 30% of patients developing metastasis and 60% dying within 1–2 years of diagnosis. Recent advancements in tumor immunology and necroptosis have provided new insights into kidney cancer therapy. Therefore, it is crucial to identify potential targets for combining immunotherapy with necroptosis.

**Materials and methods:**

Using the GSE168845 dataset and necroptosis-related genes, we identified genes that are differentially expressed in relation to necroptosis. We analyzed the prognostic value of these genes through differential expression analysis, prognostic analysis, and Cox regression analysis. The expression levels of the MYCN and CDKN2A genes were verified using the GSE53757 dataset. We also examined the association between the differentially expressed genes and clinicopathological features, as well as overall survival in our cohorts. In addition, we constructed a lasso Cox regression model to assess the correlation between these genes and immune score, ICP, and OCLR score. We conducted qRT-PCR to detect the expression of MYCN, CDKN2A, and ZBP1 in different samples of kidney renal clear cell carcinoma (KIRC). The expression levels of these genes were verified in a normal kidney cell line (HK-2 cells) and two KIRC cell lines (786-O, ACHN). The protein levels of MYCN and CDKN2A were detected using immunohistochemistry (IHC). SiRNA was used to silence the expression of MYCN and CDKN2A in the ACHN cell line, and wound healing assays were performed to measure cell migration.

**Results:**

MYCN, CDKN2A, and ZBP1 were identified as necroptosis-related genes with independent prognostic value, leading to the development of a risk prognostic model. The expression of the CDKN2A gene was significantly higher in KIRC tissues compared to normal tissues, while the expression of the MYCN gene was significantly lower in KIRC tissues. The expression of MYCN and CDKN2A was associated with tumor stage, metastasis, and overall survival in our cohort. Furthermore, MYCN, CDKN2A, and ZBP1 were significantly correlated with immune score, ICP, and OCLR score. The expression levels of CDKN2A and ZBP1 were higher in KIRC cells compared to normal kidney cells, while the expression of MYCN was lower in KIRC cells. The protein expression of MYCN and CDKN2A was also higher in KIRC tissues, as confirmed by IHC. The results of the wound healing assay indicated that silencing CDKN2A inhibited cell migration, while silencing MYCN enhanced cell migration.

**Conclusions:**

MYCN and CDKN2A are potential targets and valuable prognostic biomarkers for combining immunotherapy with necroptosis in kidney renal clear cell carcinoma. CDKN2A promotes the migration of renal cancer cells, while MYCN inhibits their migration.

## Introduction

RCC accounts for about 3% of all cancers, with the highest incidence rate in western countries. Renal cell carcinoma (RCC) is a common malignant tumor of the genitourinary system, accounting for 90% of renal malignant tumors [1]. The incidence rate of RCC has been increasing by 2% to 4% annually since 1975 [2]. RCCs comprise a broad spectrum of histological entities described in the 2016 World Health Organization (WHO) classification [3]. There are three main RCC types: ccRCC (70–80%), pRCC (types I and II, 10–15%, of which 60–70% are type I), and chromophobe RCC (4–5%). RCC aetiology includes lifestyle factors such as smoking, obesity, and hypertension. Having a first-degree relative with RCC is also associated with higher risk. Other factors include specific dietary habits, diabetes, and occupational exposure to specific carcinogens, but the literature is inconclusive. Preventative measures include elimination of cigarette smoking and reducing obesity [4–6]. Many scholars have studied the diagnosis and treatment technology of KIRC and achieved a series of results, but the prognosis of KIRC patients is still poor and there is much room for improvement [7]. The proportion of tumor recurrence has reached one third [8, 9]. The survey results showed that in 2020, about 179368 patients died of RCC, and the number of new cases reached 124578 [10]. At present, the research on the pathological mechanism of KIRC is still not in-depth, and the corresponding high-sensitivity biomarkers are still missing. Therefore, it is necessary to develop effective therapeutic targets or sensitive prognostic markers for KIRC.

Necrosis and apoptosis are two classical types of cell death. The former is a passive and uncontrollable cell death. Severe membrane damage leads to rapid redistribution of cell components and high swelling of cells, resulting in cell rupture and release of DAMPs [11]. Apoptosis is caspase dependent programmed cell death [12]. Nowadays, with the in-depth study of cell death mechanism, many new types of cell death have been found, such as iron death, oncosis, apoptosis like and necrotic apoptosis. As a non-caspase dependent programmed cell death with necrotic characteristics, necrotic apoptosis has attracted extensive attention in many systematic disease studies. The concept of necroptosis originated from the study of death receptors. Caspase is a key regulator of apoptosis, but some scholars found that when tumor necrosis factor receptor (TNFR) is activated, inhibiting caspase cannot prevent cell death, but transfer cells to a death mode with necrotic morphological characteristics [13]. Degterev et al. [14] screened Necrostatin-1 (nec-1), a specific inhibitor of this death type, and named this special form of cell death necroptosis for the first time. Subsequent studies further confirmed that necrotic apoptosis was mainly mediated by the activation of RIP1, RIP3 and MLKL [15, 16].

A large number of experimental results show that necroptosis plays two regulatory roles in the pathological process of cancer [17], which is mainly related to the expression level of targeted necrosis protein [18]. The dysfunction of necrotic ptosis is closely related to the pathological process of tumor. A typical example is that in patients with acute myeloid leukemia (AML), the expression level of RIP3 decreases, which inhibits the differentiation and apoptosis of hematopoietic cells, which is closely related to the occurrence of AML [19]. It can be inferred that the low expression of MLKL generally indicates poor prognosis of patients [20].

As a new way of cell death, necroptosis participates in the development of cancer and plays a role in many tumors. Strilic et al. [21] found that tumor cells cause necroptosis of endothelial cells. In malignant glioma, RIPK1 causes poor prognosis [22]. RIPK1/3 can also play a role in renal cancer [23]. In VHL deficient renal clear cell carcinoma, cystine deprivation can cause programmed necrosis of tumor cells [24]. Al Lamki et al. [25] found that the expression level of RIPK1/3 obviously enhanced in renal clear cell carcinoma and was prone to necroptosis when TNFR1 signaling pathway was activated. Thus, as a possible death mode of tumor cells in such carcinoma, the level of necroptosis related genes and potential regulatory axis may have implications for the prognosis of patients.

However, the function and mechanism of necrosis in KIRC are still not clear, and there are many problems to be further explored. In this context, this paper studied the expression changes of necrosis-related genes in normal kidney and KIRC tissues and studied the prognostic value of these genes and their correlation with TME, so as to provide a basis for the treatment of KIRC and play a reference role for the same type of research.

## Materials and methods

### Microarray data analysis and screening of necroptosis-related differentially expressed genes

To identify necroptosis-related DEGs in kidney renal clear cell carcinoma (KIRC), we utilized the Gene GEO database and selected the GSE168845 dataset for further analysis. The necroptosis-related gene set M24779 gmt, consisting of 8 necroptosis-related genes, was downloaded from the gene set enrichment analysis (GSEA). In accordance with previous reports, we identified 67 necroptosis-related genes and constructed a gene map.

The DEGs were considered statistically significant if they met the cut-off criteria of adjusted P < 0.05 and |log2FC|≥ 1. Volcano plots and Venn diagrams were generated using ImageGP.

### Functional enrichment analysis of necroptosis-related-DEGs in KIRC

To explore functional annotation and enrichment pathways, we performed gene ontology (GO) and Kyoto Encyclopedia of Genes and Genomes (KEGG) pathway analyses using the ClusterProfiler software package. Statistically significant differences were defined as p < 0.05.

### Survival analysis and verification

To further evaluate the expression and prognostic value of necroptosis-related-DEGs in KIRC, we conducted differential analysis and prognostic analysis using the survival package. The risk ratio (HR) was calculated based on Cox proportional hazards model and Kaplan–Meier model, with p < 0.05 indicating statistically significant differences.

### Construction and validation of the necroptosis-related-DEGs prognostic model

Following the preliminary screening of DEGs with differential expression and prognostic significance, univariate Cox analysis of overall survival (OS) was performed to identify survival-related DEGs with significant prognostic value (p < 0.05). Subsequently, multivariate Cox regression analysis was conducted to construct a prediction model based on these DEGs, which were found to be independent prognostic factors. LASSO regression, known for variable selection and complexity adjustment, was utilized to model and predict. This technique allowed for the selection of variables that improved performance parameters rather than fitting all variables into the model. Complexity adjustment was achieved by controlling model complexity through a series of parameters to prevent overfitting [26, 27]. Signatures were established based on the coefficients corresponding to the independent prognostic genes. Patients from the TCGA-KIRC dataset were divided into low and high-risk groups based on the risk score obtained from the multivariate Cox regression. R packages such as t-distributed stochastic neighbor embedding (t-SNE) and principal-component analysis (PCA) were employed to explore the distribution characteristics of different groups. The effectiveness of prognostic indicators was evaluated by the area under the curve (AUC) of the ‘‘time receiver operating characteristic curve (ROC)’’.

### Validation of the necroptosis-related-DEGs

To validate the expression of MYCN and CDKN2A, we utilized the GSE53757 and GSE105261 datasets. The pathology of KIRC patients was confirmed by experienced doctors, and the latest version of the United States Joint Commission on Cancer TNM staging system was used for pathological staging. We analyzed the correlation between DEGs and clinicopathological characteristics, as well as overall survival in our cohorts.

### Clinicopathological correlation and determination of nomogram

To explore the relationship between DEGs and clinicopathological characteristics, we utilized the “survival” software package in R and consulted with doctors and experts. Based on this, we created a nomogram and calibration curve using the R package ‘‘rms’’. Additionally, we introduced a classic prognostic model to reflect changes in overall survival over different time periods.

### GSEA enrichment analysis

The cluster analyzer package was employed to efficiently perform GSEA on DEGs. Statistical significance was determined if the calculated value was less than 0.05.

### Relationship between DEGs and immune microenvironment

We introduced the xCell algorithm for “immune de-noising” and discussed the correlation between DEGs and immune cells, taking into consideration relevant literature. Furthermore, using data and information from the “ggplot2” R package, we determined the correlation between gene expression and genes related to eight immune checkpoints. After completing the above analysis, we utilized the TIDE algorithm to investigate and reveal the immune escape principles of human tumors in relation to DEGs markers.

### Cell lines, patients samples, RNA extraction

The human kidney cell lines HK-2, 786-O and ACHN used in this study are from the Shanghai Institute of Life Sciences. After collecting the cell sample, put it into 1640 medium (GIBCO) for culture, which contains fetal bovine serum, streptomycin, etc. During this process, it is necessary to maintain 5% carbon dioxide content.

In this paper, a total of 25 fresh samples were selected, and then according to the research needs, the KIRC samples removed by patients in recent years were obtained, and then stored in a high-temperature environment. All patients were diagnosed as KIRC by doctors with rich clinical experience, and all subjects were not given anti-tumor treatment recently. The research in this paper is carried out in accordance with the Helsinki Declaration and has been approved by relevant authorities. All subjects knew the contents and methods of this study, and then signed the informed consent form. Our study is retrospective.

The total RNA kit used in this study was separated according to the manufacturer's instructions. In addition, the reagent kit (vazyme) is also used. In this paper, the classical 2 − ΔΔCt method. is introduced to complete the normalization of the relevant data, so that the expression of GAPDH can be obtained.

### Tissue microarray construction and immunohistochemistry (IHC)

The samples used in this paper are fixed in formaldehyde solution and then embedded in paraffin. Then process the samples obtained in the above steps with DAB color method. Primary antibody (MYCN, ab198912, Abcam) and (CDKN2A, ab54210, Abcam) were used in this link.

### SiRNA interference and cell grouping

ACHN cells were cultured in RPMI-1640 medium (containing 10% fetal bovine serum) in an incubator at 37 ℃, with a volume fraction of 5% CO2 and saturated humidity until the cell fusion degree was 70–80%. 0.25% trypsin was added for digestion and subcultured at a ratio of 1:2. Take ACHN cells of renal cell carcinoma in logarithmic growth phase, with 1 × 10^5^ pieces/mL were inoculated onto a 96 well culture plate and randomly divided into two groups: the experimental group and the negative group. The experimental group cells were transfected with CDKN2A or MYCN inhibitor and Negative control according to the instructions of Lipfectamine 2000 liposome transfection kit, respectively. After transfection for 4 h, replace the culture medium and continue to culture for 48 h. Collect logarithmic growth phase cells from each group for subsequent experiments.

### Wound healing assay

Inoculate 5 cells (ACHN) per well in 6-well plateX10^5^ cells, after the cells grew to 90% density, 200 μL gun head was used to scratch the cells. After washing with PBS until there were no obvious floating cells, the medium was changed to serum-free RPMI-1640 medium, and fixed-point photographs were taken at 0 and 24 h. Then use the powerful image J software to further determine the scratch area, and then enter the next step of research.

### Statistical analysis

For the data obtained from the study, the latest version of R software is mainly used for statistics. All indicator data are processed with powerful Perl programming language. For the prognosis, further multivariate Cox regression analysis is needed. Finally, the value of p is calculated. If the value is less than 0.05, it means that the data are significantly different. Fellow researchers can reproduce my experiments through my methods. The number of experiments that we conducted is three times.

## Results

### Identification of necroptosis related DEGs in KIRC comparing to normal renal tissues

**Fig. 1:**
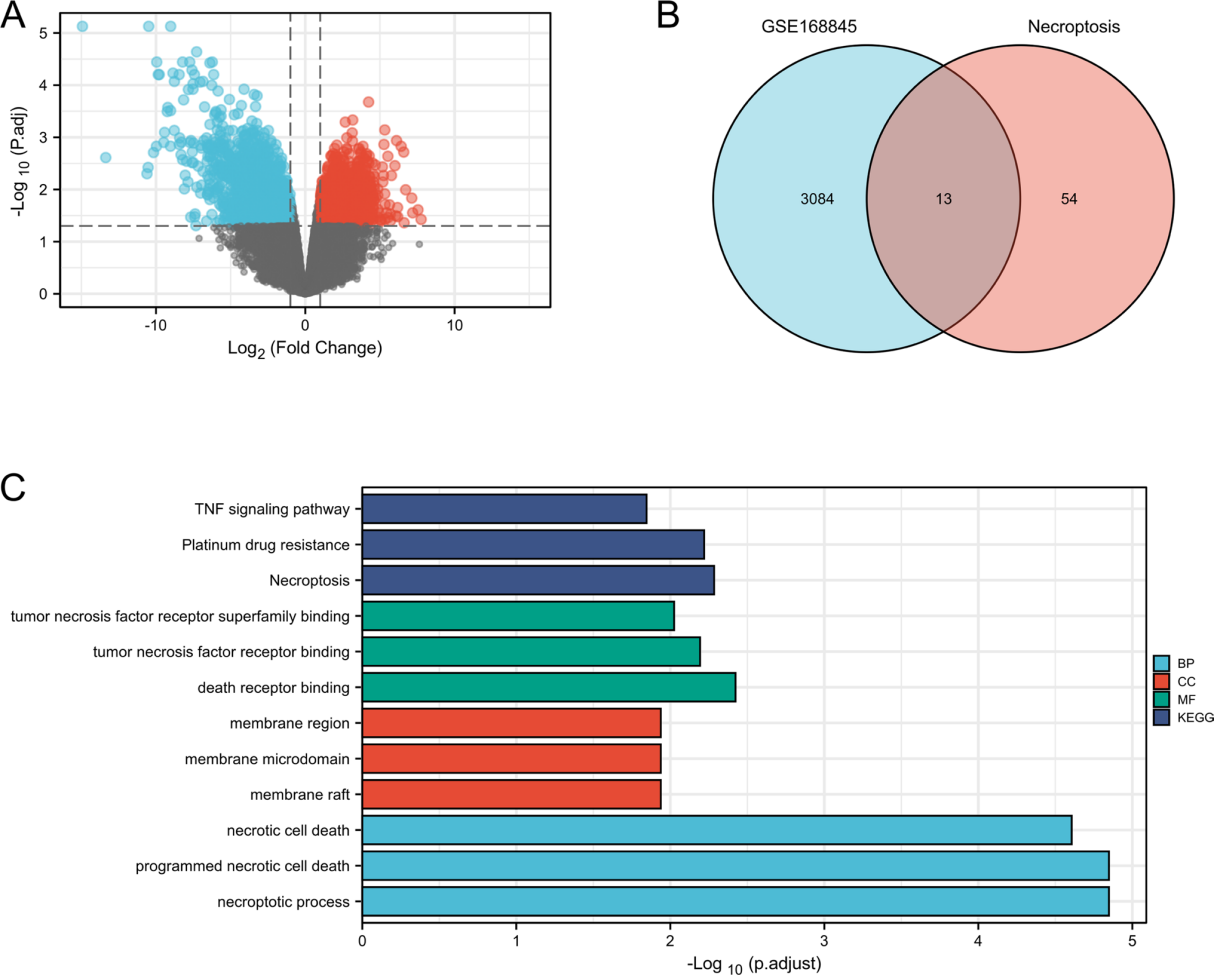
Volcano plots of DEGs between healthy renal tissues and renal cancer in GSE168845 samples **A**. Adjusted P-value < 0.05 and log2-FC > 1. 3097 DEGs were screened with 1288 upregulated genes and 1809 downregulated genes. And they are represent by red and blue lattice, respectively. Up to 67 necroptosis-related genes were obtained. Venn diagram showing the 13 necroptosis genes based on three datasets **B**. Graph reflected the GO and KEEG analysis of the 13 necroptosis genes **C**. The 13 necroptosis genes were TNFRSF1B, FASLG, MYCN, CASP8, BACH2, CDKN2A, GATA3, IPMK, MLKL, AXL, ID1, CD40 and ZBP1

The volcanic map visually shows 1288 up-regulated DEGs selected by the research team, and 1809 down-regulated DEGs, as shown in Fig. 1A. In this link, 67 necrosis-related genes were also objectively reflected by Venn map, and on this basis, 13 co-expression genes were further identified: TNFRSF1B, FASLG, MYCN, CASP8, BACH2, CDKN2A, GATA3, IPMK, MLKL, AXL, ID1, CD40 and ZBP1 (Fig. 1B). In the analysis of GO and KEGG, it turned out that the functions of the 13 co-expressed genes were mainly focused on “necroptosis process”, “death receptor binding” and “necroptosis” (Fig. 1C).

### Differential expression analysis and survival analysis of necroptosis related DEGs in KIRC

**Fig. 2:**
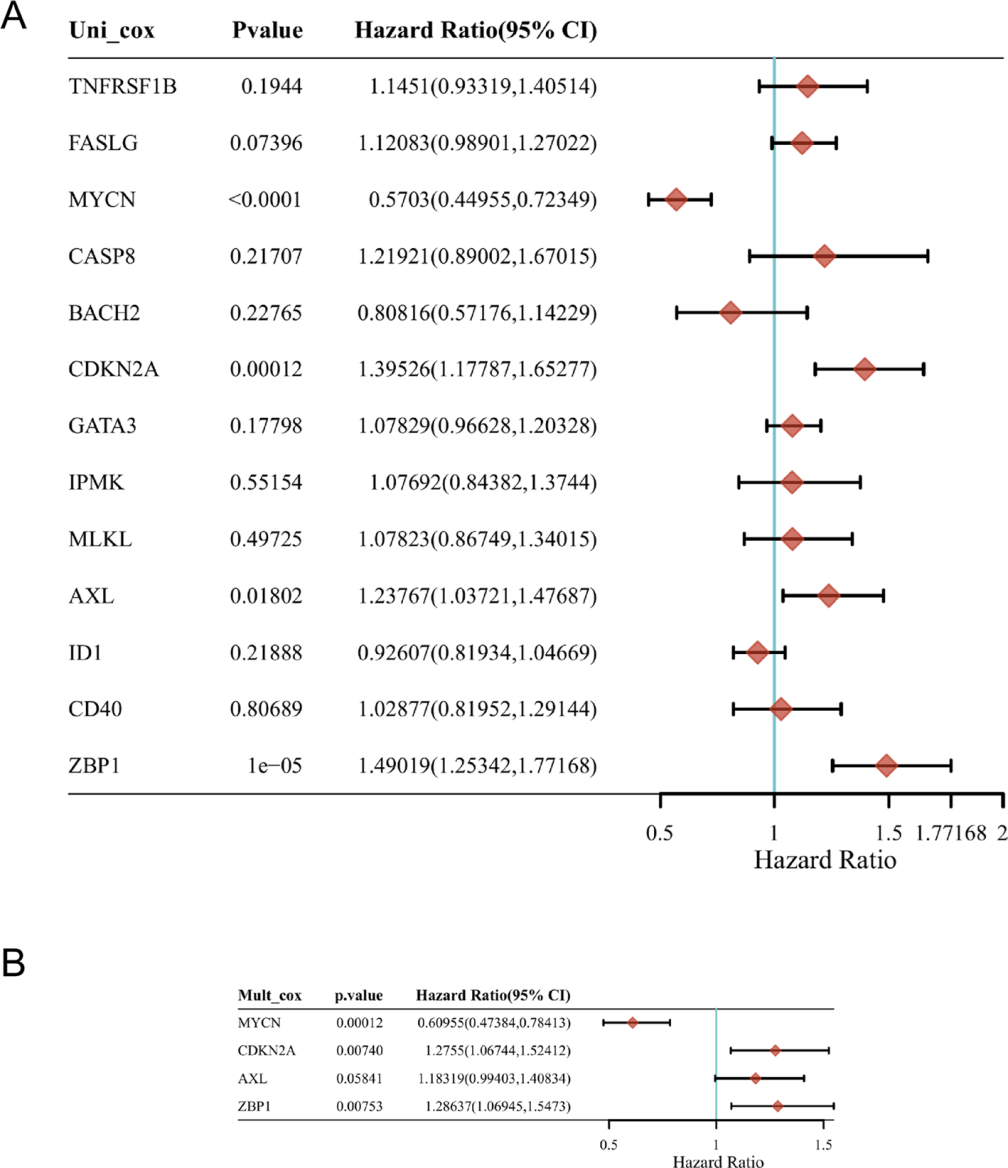
The forest plot showing the results of the univariate Cox regression analyses of the 13 necroptosis genes in TCGA-KIRC **A**. That of the 4 necroptosis genes in TCGA-KIRC **B**. And MYCN, CDKN2A and ZBP1 were significant

In this paper, single-factor and multi-factor Cox regression analysis is introduced to summarize the impact of 13 DEGs on the prognosis. The conclusion is that MYCN and CDKN2A can affect KIRC (Fig. 2A–B).

**Fig. 3:**
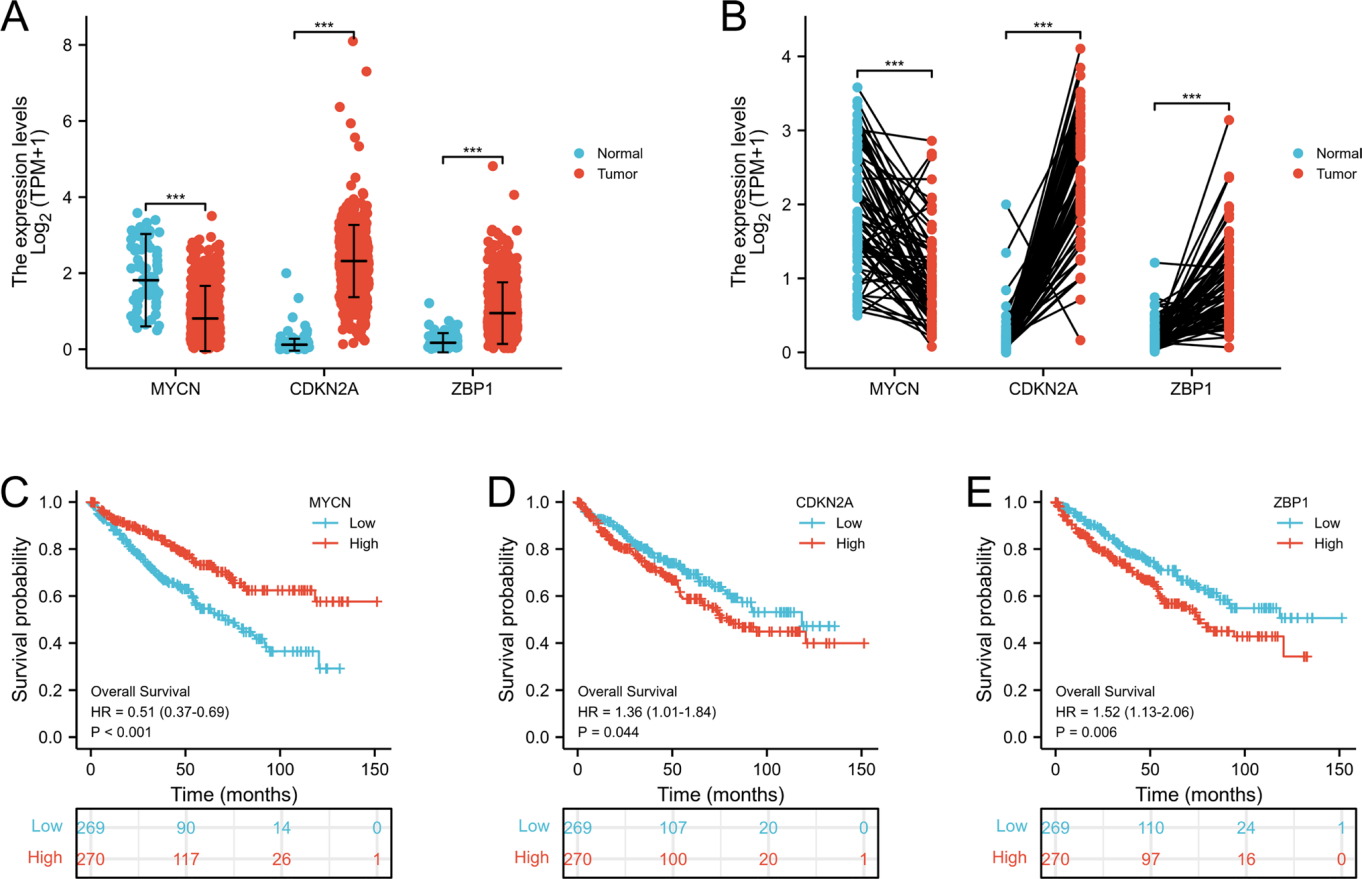
Expression profile of the 3 DEGs in KIRC samples compared with normal tissues **A**–**B**. Kaplan–Meier plots showing MYCN, CDKN2A and ZBP1with prognostic value **C**–**E**. $$*\mathrm{p}<0.05, **\mathrm{p}<0.01, ***\mathrm{p}<0.001$$

After collecting and processing the data in TCGA-KIRC database, the researchers compared the content of MYCN and other factors in normal kidney tissue and pathological tissue and found CDKN2A and ZBP1 in tumor tissues were up-regulated and MYCN was down-regulated (Fig. 3A–B). Kaplan–Meier model analysis showed that the increased content of MYCN would enhance the prognosis effect (Fig. 3C). On the contrary, the increased content of CDKN2A and ZBP1 in tissues would reduce the prognosis effect, and the two were closely related (Fig. 3D–E).

### Necroptosis related gene DEGs GSEA analysis

**Fig. 4:**
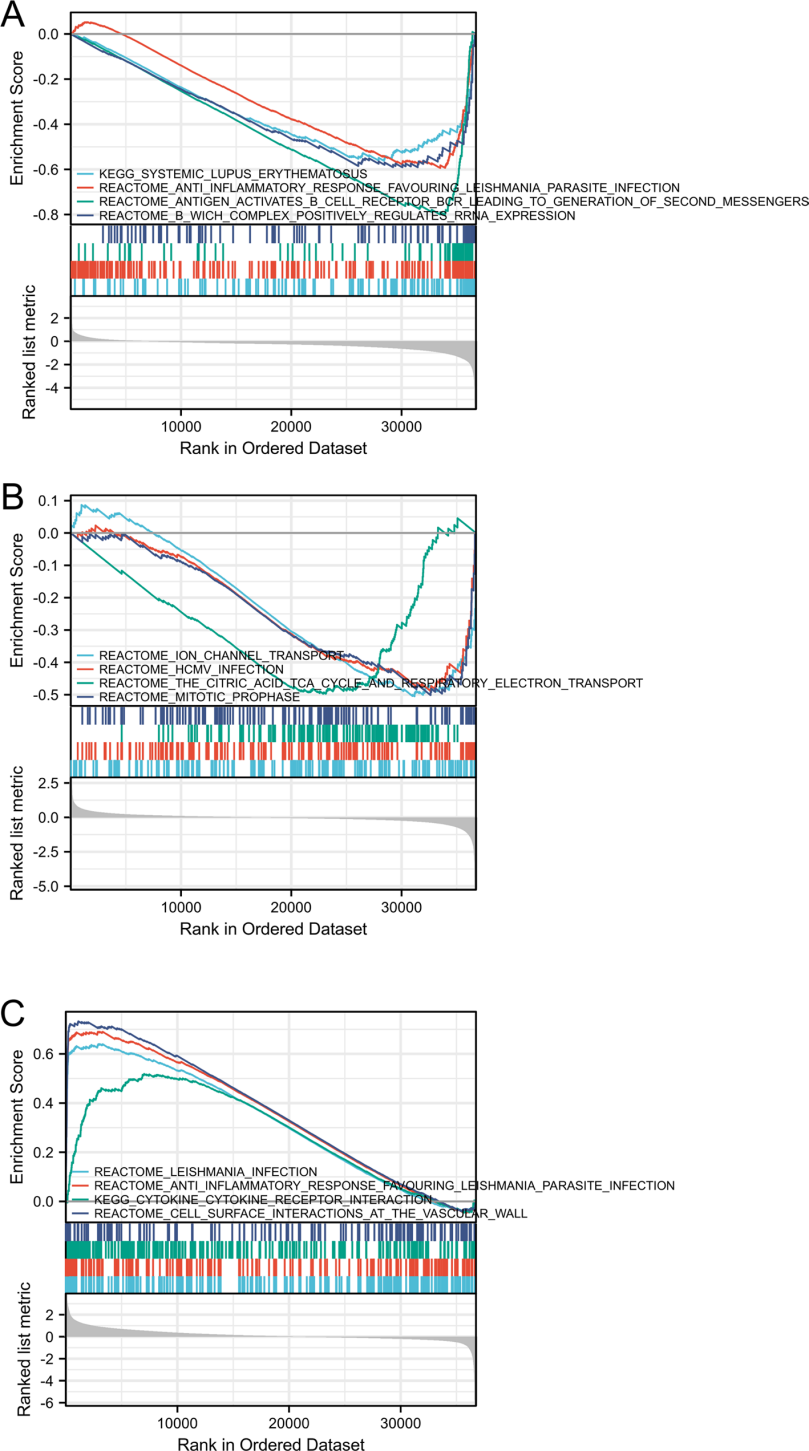
Necroptosis Related Gene DEGs GSEA analysis. Enrichment analysis of MYCN **A**, CDKN2A **B** and ZBP1 **C**

We used collected a large number of KIRC patient data in the TCGA-KIRC database, and then used GSEA to conduct in-depth analysis of these data. The conclusion is that MYCN mediated anti-inflammatory response (Fig. 4A), CDKN2A mediated ion-channel transport (Fig. 4B) and ZBP1 mediated leishmania-infection (Fig. 4C).

### Construction and validation of the necroptosis related DEGs prognostic risk model

**Fig. 5:**
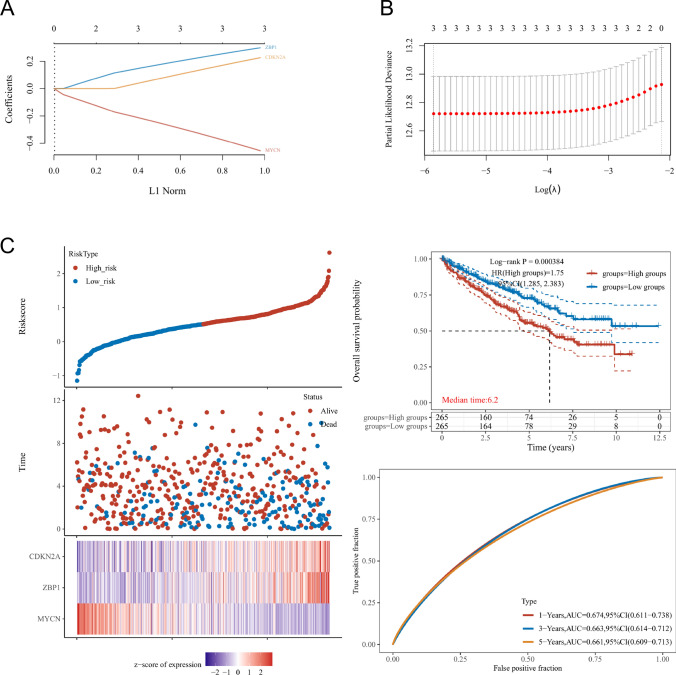
The calculations for the model based on Cox regression result **A**–**B**. The prognostic model was analyzed by survival time, status, heat map, and 1/3/5 year OS **C**. lambda.min = 0.0029. Riskscore = (− 0.4545) *MYCN + (0.2261)*CDKN2A + (0.2984)*ZBP1

Use lasso cox regression to further build the risk prognosis model of DEGs, lambda.min = 0.0029, Riskscore = (− 0.4545) *MYCN + (0.2261) *CDKN2A + (0.2984) *ZBP1 (Fig. 5A–B). Based on the median risk score, the subjects were divided into high-risk group and low-risk group. After statistics, the HR is 1.75, and the prognosis model can be directly used to replace the risk factor model. After statistical processing of various data, it is concluded that the median survival time of the high-risk group is lower in the two groups, and the data difference between the groups is statistically significant (Fig. 5C). In this link, ROC is also introduced to analyze the prognostic efficiency of the above models. The results are shown in Fig. 5C.

### Relationship between necroptosis related DEGs and clinicopathological factors

**Fig. 6:**
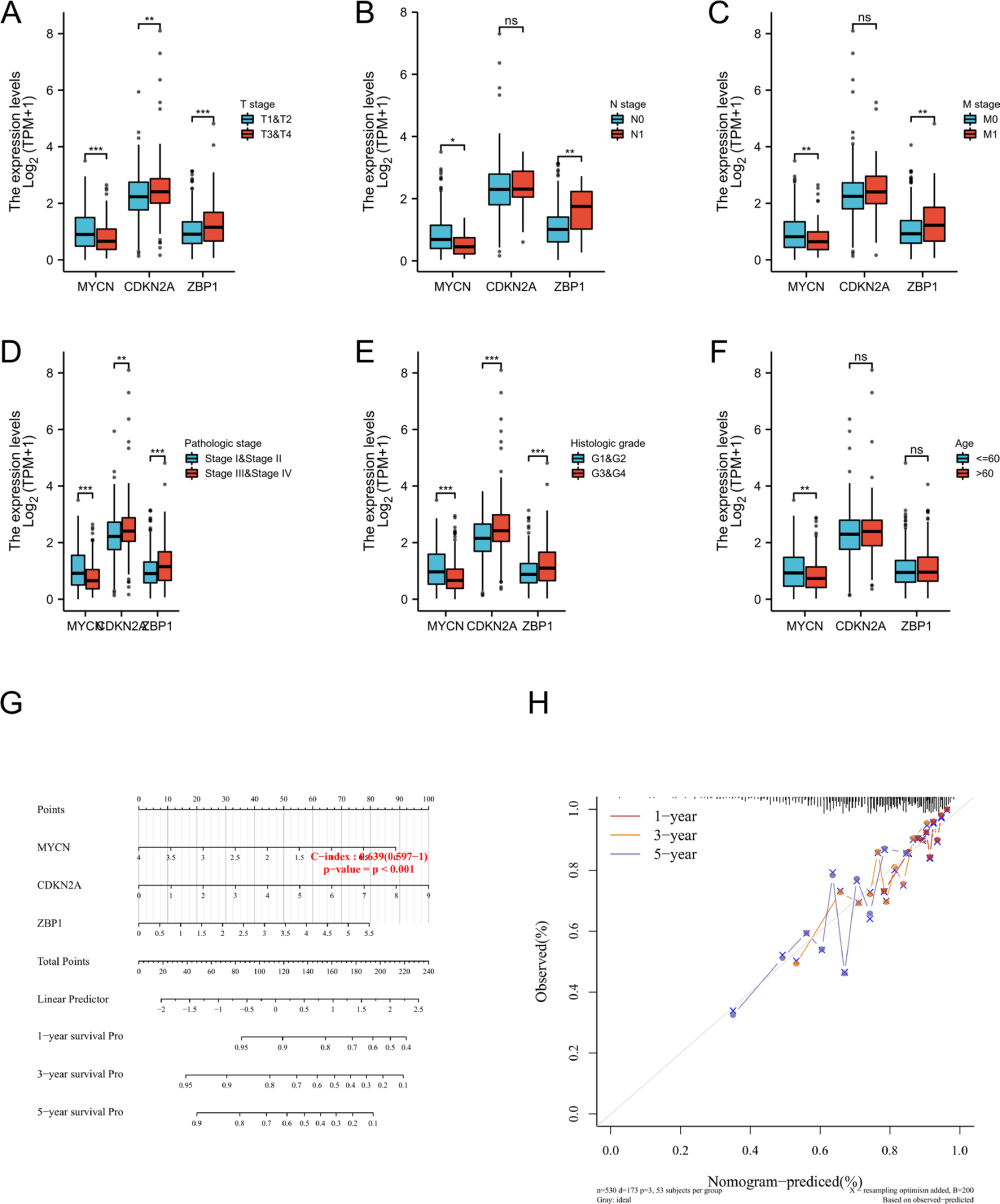
The 3 necroptosis genes obviously correlate with clinicopathological factors in KIRC patients. The association between MYCN, CDKN2A, ZBP1 in entire TCGA cohort **A**–**F**. Nomogram for predicting 1‐, 3‐, and 5 year OS in entire TCGA cohort **G**. Calibration curves of nomogram on consistency between predicted and observed 1-, 3-and 5-year OS in entire TCGA cohort (**H**). Dashed line at 45° implicated a perfect prediction.$$*\mathrm{p}<0.05, **\mathrm{p}<0.01, ***\mathrm{p}<0.001$$

After analyzing the risk prognosis model, the research group found that necrosis-related DEGs were closely related to the pathological stage, but not to age (Fig. 6A–F). Use the nomograph to predict the OS of the entire TCGA queue in different time periods, with the c index of 0.639 (Fig. 6G). We also found that the 1 year, 3 year and 5 year OS on the nomograph converged to the corresponding predicted probability calibration curve (Fig. 6H).

### Correlation between the expression of immune infiltrating cells in KIRC tissues and necroptosis related DEGs

**Fig. 7:**
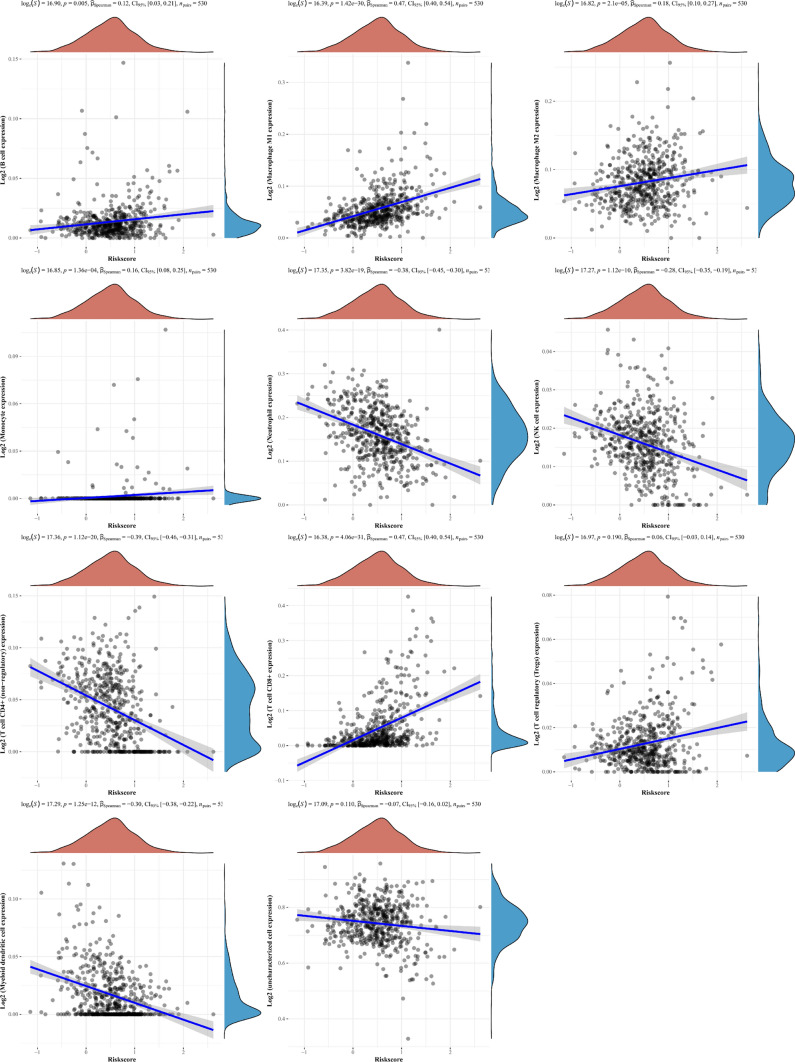
Spearman correlation result of risk score and immune score

Spearman correlation analysis between prognosis model score and immune score was made in Fig. 7. The results showed that the prognosis model could directly affect the content of various immune infiltrating cells in tissues, such as B cells, macrophages M1 cells, macrophages M2 cells, monocytes, CD8 + T cells, etc.

**Fig. 8:**
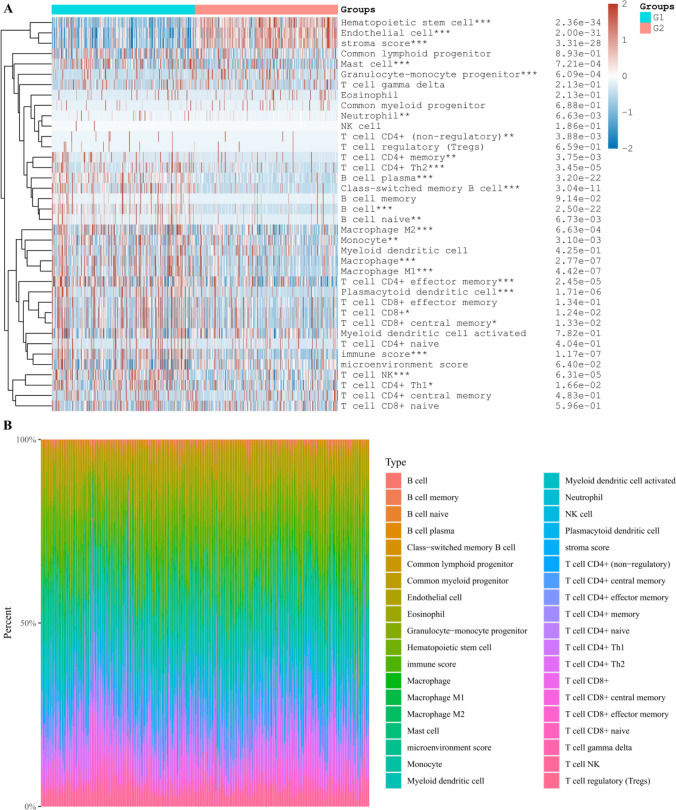
Differential proportions of infiltrating immune cells in kidney renal clear cell carcinoma (KIRC) tissues from patients with low or high expression levels of MYCN gene **A**. The percentage abundance of infiltrating immune cells in renal clear cell carcinoma, with different colors representing different types of immune cells, horizontal axis representing samples, and vertical axis representing the percentage of immune cell content in a single sample **B**. G1 and G2 are low and high expression groups, respectively. *p < 0.05, **p < 0.01, ***p < 0.001

**Fig. 9:**
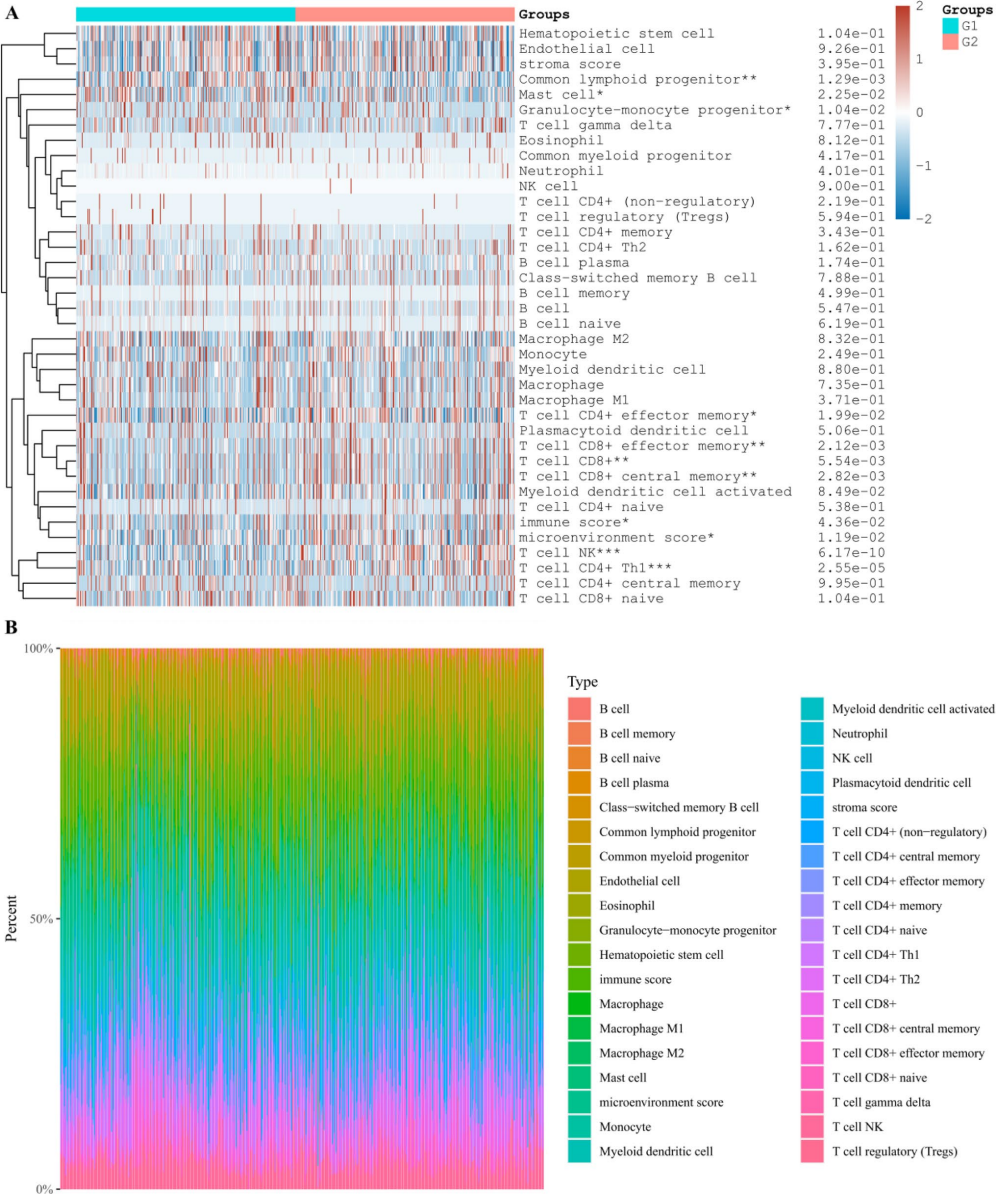
Differential proportions of infiltrating immune cells in kidney renal clear cell carcinoma (KIRC) tissues from patients with low or high expression levels of CDKN2A gene **A**. The percentage abundance of infiltrating immune cells in renal clear cell carcinoma, with different colors representing different types of immune cells, horizontal axis representing samples, and vertical axis representing the percentage of immune cell content in a single sample **B**. G1 and G2 are low and high expression groups, respectively. *p < 0.05, **p < 0.01, ***p < 0.001

**Fig. 10:**
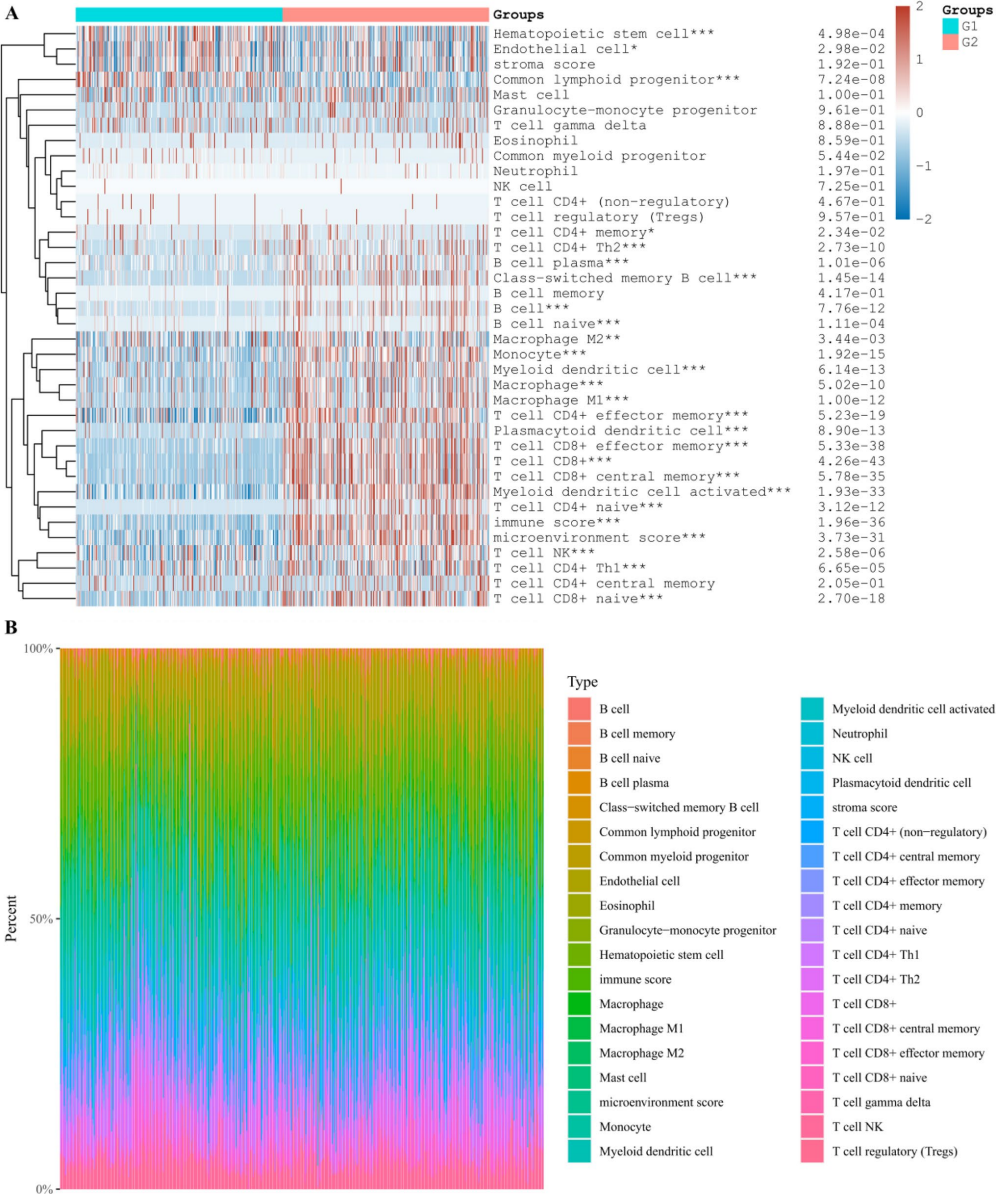
Differential proportions of infiltrating immune cells in kidney renal clear cell carcinoma (KIRC) tissues from patients with low or high expression levels of ZBP1 gene **A**. The percentage abundance of infiltrating immune cells in renal clear cell carcinoma, with different colors representing different types of immune cells, horizontal axis representing samples, and vertical axis representing the percentage of immune cell content in a single sample **B**. G1 and G2 are low and high expression groups, respectively. *p < 0.05, **p < 0.01, ***p < 0.001

After collecting and processing the data in the TCGA-KIRC database, the KIRC population was divided into groups, namely, the low expression group (G1) and the high expression group (G2) of DEGs, and the correlation between the amount of immune infiltrating cells and DEGs was further discussed. It can be seen that in the body, MYCN and CDKN2A will affect the content of various immune infiltrating cells to varying degrees. Further analysis shows that the content of haemophile stem cells and B cells in the tissue will affect the level of MYCN and CDKN2A (Figs. 8, 9, 10). It is speculated that the above cells may affect the progress of KIRC.

### Correlation between the expression of immune checkpoint in KIRC tissues and necroptosis related DEGs

**Fig. 11:**
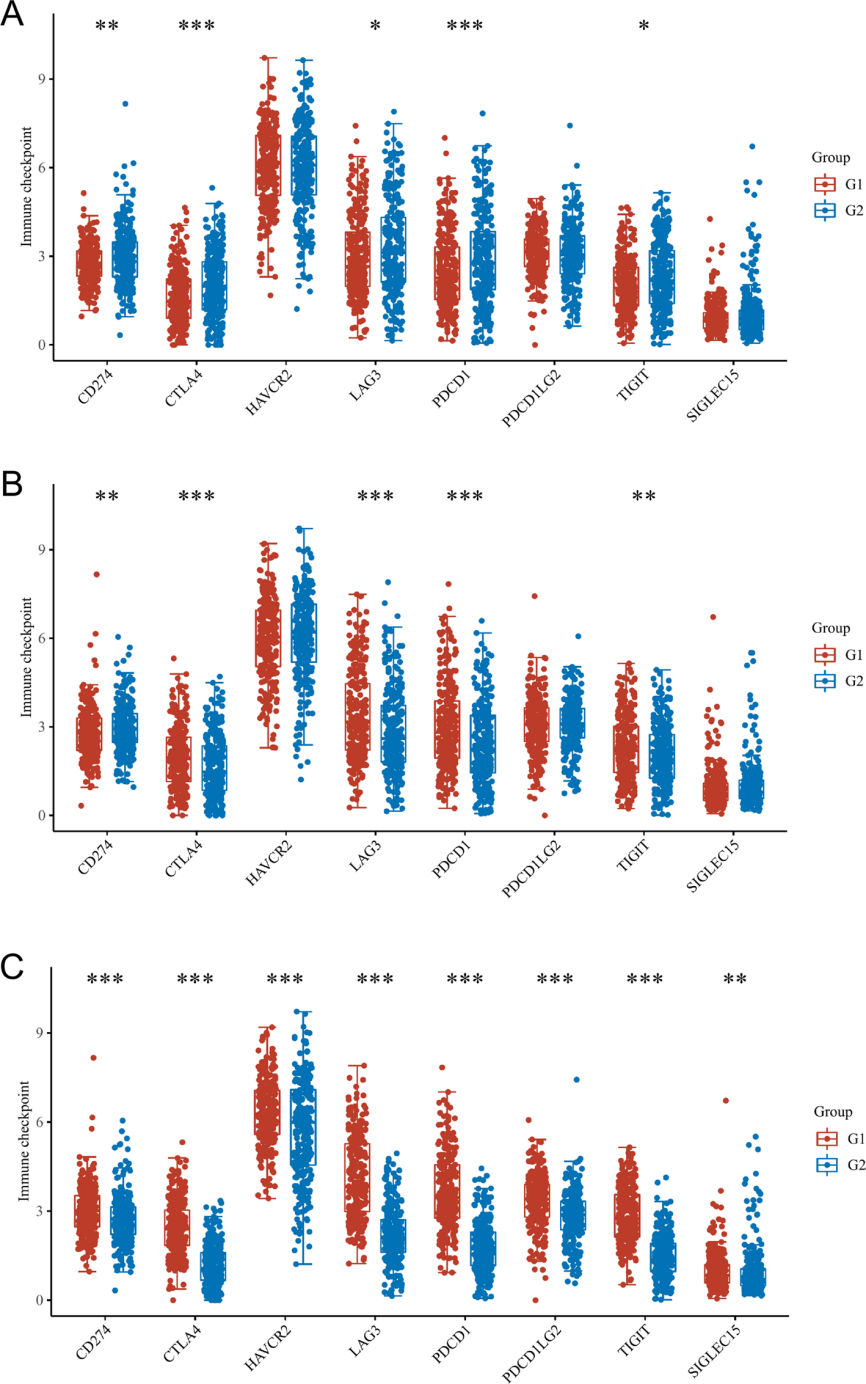
The difference of expression of immune checkpoint in KIRC tissues with various MYCN **A**, CDKN2A **B** and ZBP1 **C** gene expression. G1 is a high expression group and G2 is contrary. $$*\mathrm{p}<0.05, **\mathrm{p}<0.01, ***\mathrm{p}<0.001$$

This article mainly discusses the application value of KIRC targeted drugs. In this process, it is also necessary to determine whether the immune checkpoints in KIRC tissue will affect the expression level of necrosis related DEGs. We found that CD274, CTLA4, LAG3, PDCD1, TIGIT are closely related to MYCN and CDKN2A (Fig. 11A–B); At the same time, it is also found that CD274, CTLA4 and other factors have a close relationship with ZBP1 (Fig. 11C). It is not difficult to find thatCD274, CTLA4, LAG3, PDCD1, TIGIT appear three times. This means that they may be sensitive immune checkpoints during KIRC intervention.

**Fig. 12:**
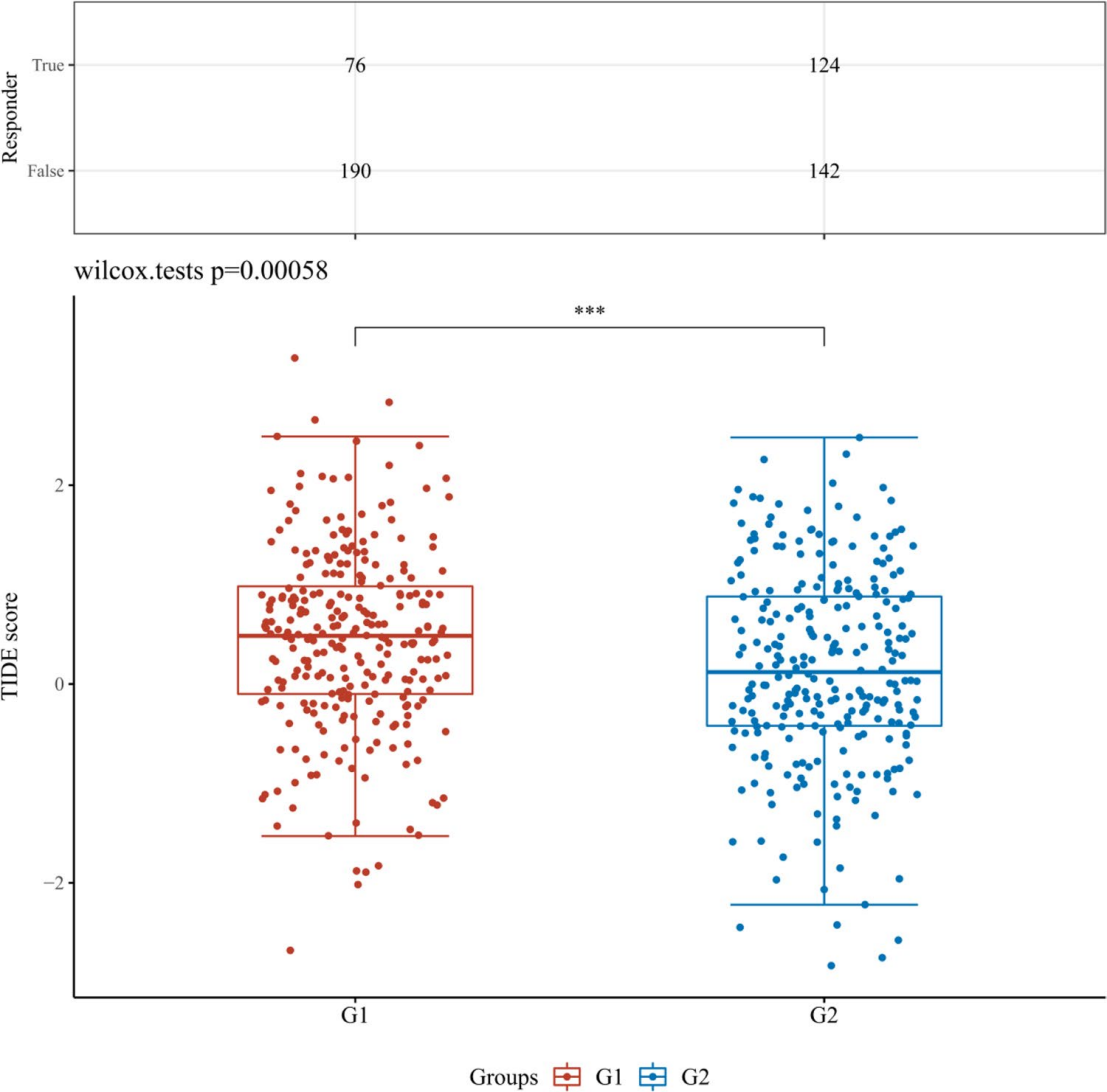
The differential immune checkpoint blockade response in KIRC tissues from patients with high or low expression levels of MYCN gene. G1 and G2 are high and low expression groups, respectively. *p < 0.05, **p < 0.01, ***p < 0.001

**Fig. 13:**
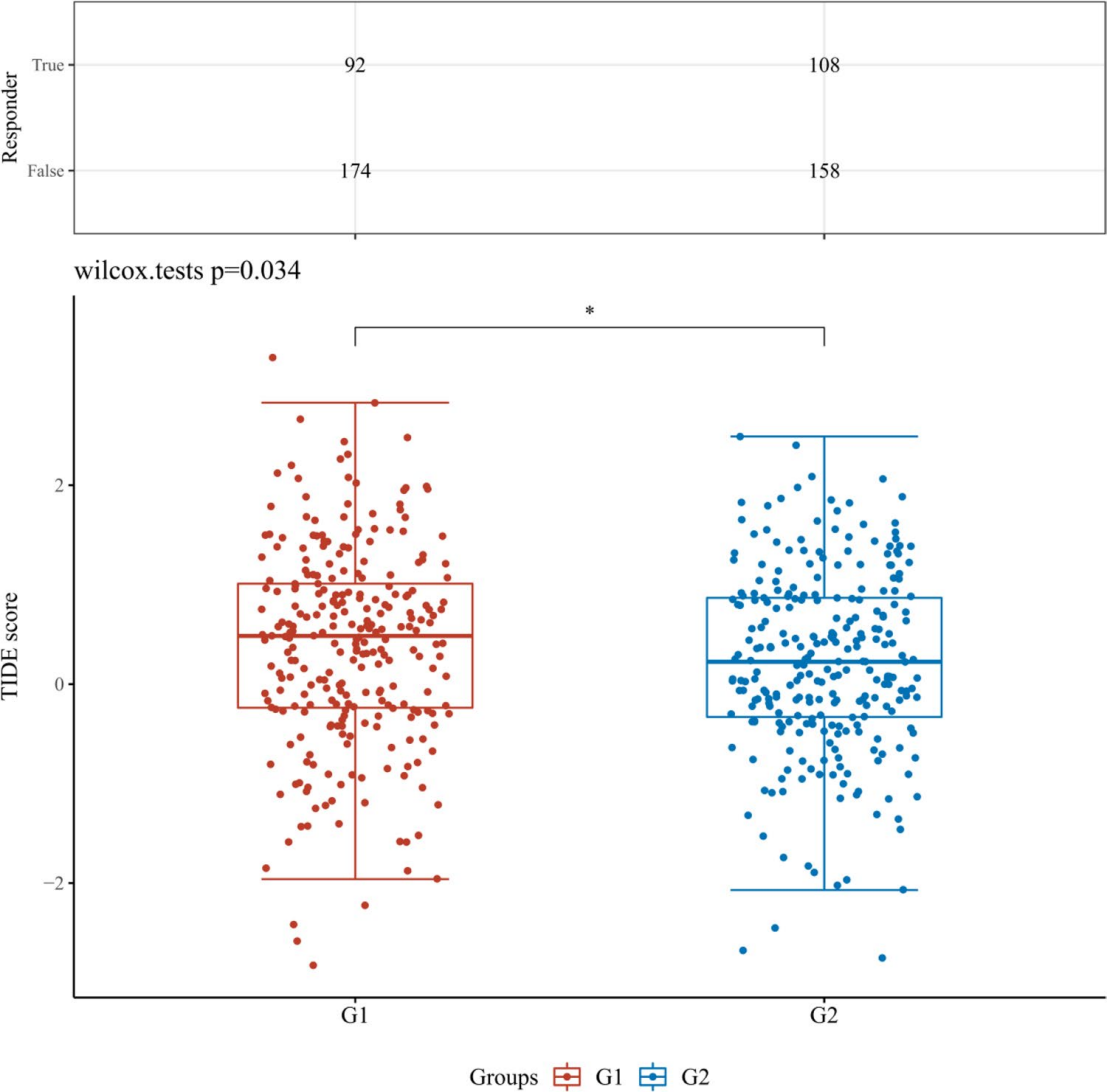
The differential immune checkpoint blockade response in KIRC tissues from patients with high or low expression levels of CDKN2A gene. G1 and G2 are high and low expression groups, respectively. *p < 0.05, **p < 0.01, ***p < 0.001

**Fig. 14:**
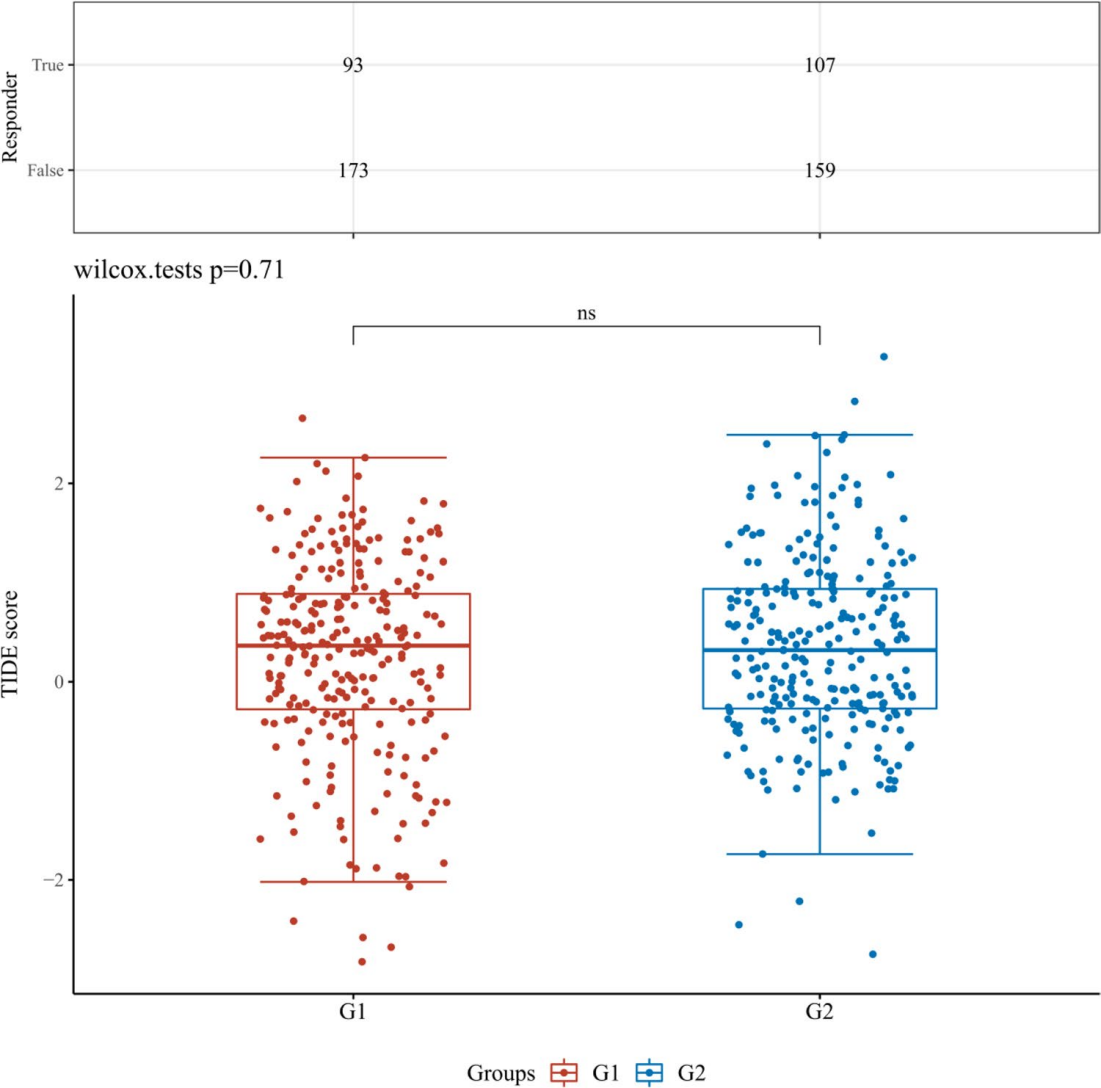
The differential immune checkpoint blockade response in KIRC tissues from patients with high or low expression levels of ZBP1 gene. G1 and G2 are high and low expression groups, respectively. *p < 0.05, **p < 0.01, ***p < 0.001

**Fig. 15:**
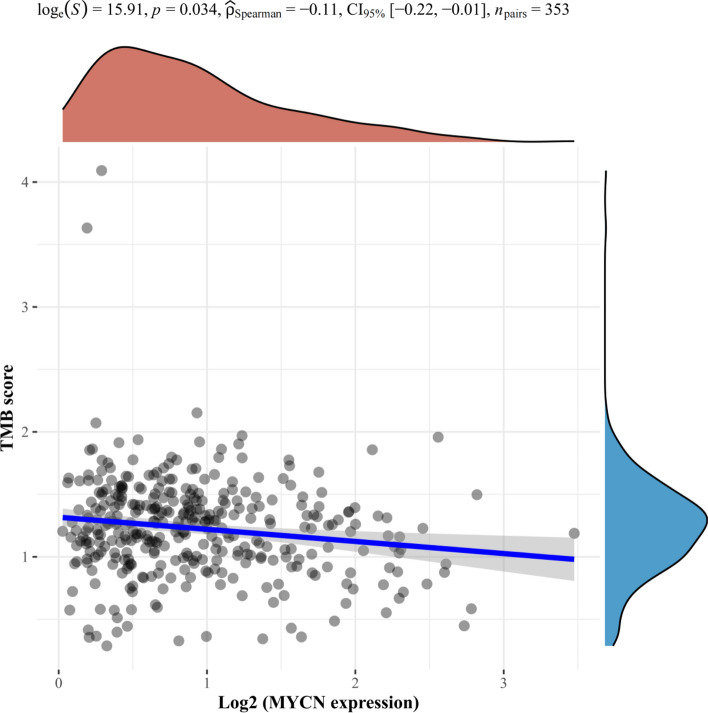
Spearman result of TMB and MYCN gene expression. The horizontal axis in the figure represents the expression distribution of genes, the vertical axis represents the distribution of TMB scores, and the density curve on the right represents the distribution trend of TMB scores; The upper density curve shows the distribution trend of gene expression; The top numerical value represents the correlation p-value, correlation coefficient and correlation calculation method

This paper also introduces the classic exclusion (TIDE) algorithm to predict the sensitivity of different levels of MYCN, CDKN2A and other indicators in tissues to immune checkpoint inhibitors (Figs. 12, 13, 14, 15). After a series of statistical analysis, it is concluded that the p value of CDKN2A and MYCN is less than 0.05, which means that immune checkpoint inhibitors can treat KIRC and have positive significance in improving the survival rate of patients.

### Correlation between tumor mutation burden and expression of necroptosis related DEGs

**Fig. 16:**
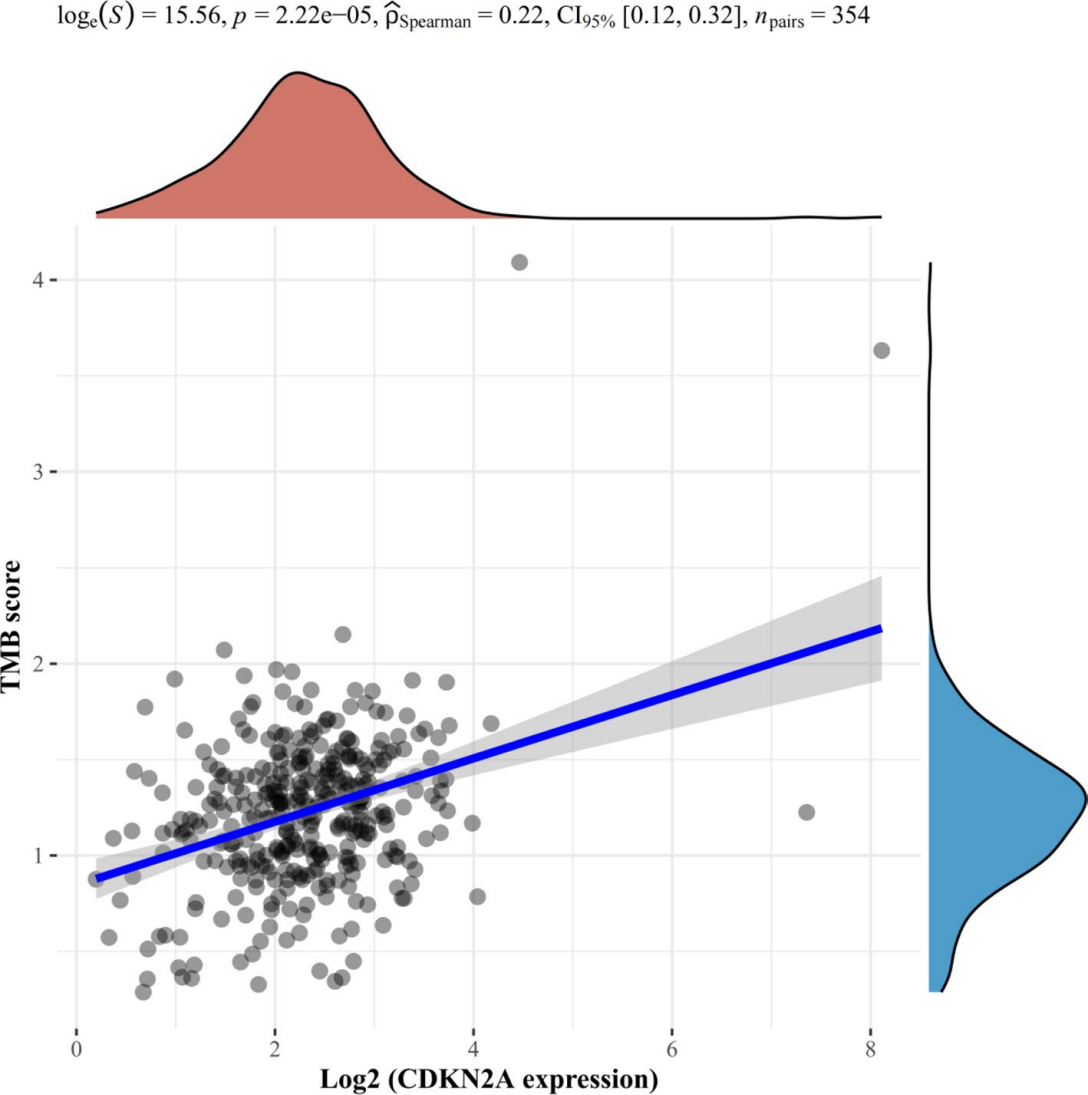
Spearman result of TMB and CDKN2A gene expression. The horizontal axis in the figure represents the expression distribution of genes, the vertical axis represents the distribution of TMB scores, and the density curve on the right represents the distribution trend of TMB scores; The upper density curve shows the distribution trend of gene expression; The top numerical value represents the correlation p-value, correlation coefficient and correlation calculation method

**Fig. 17:**
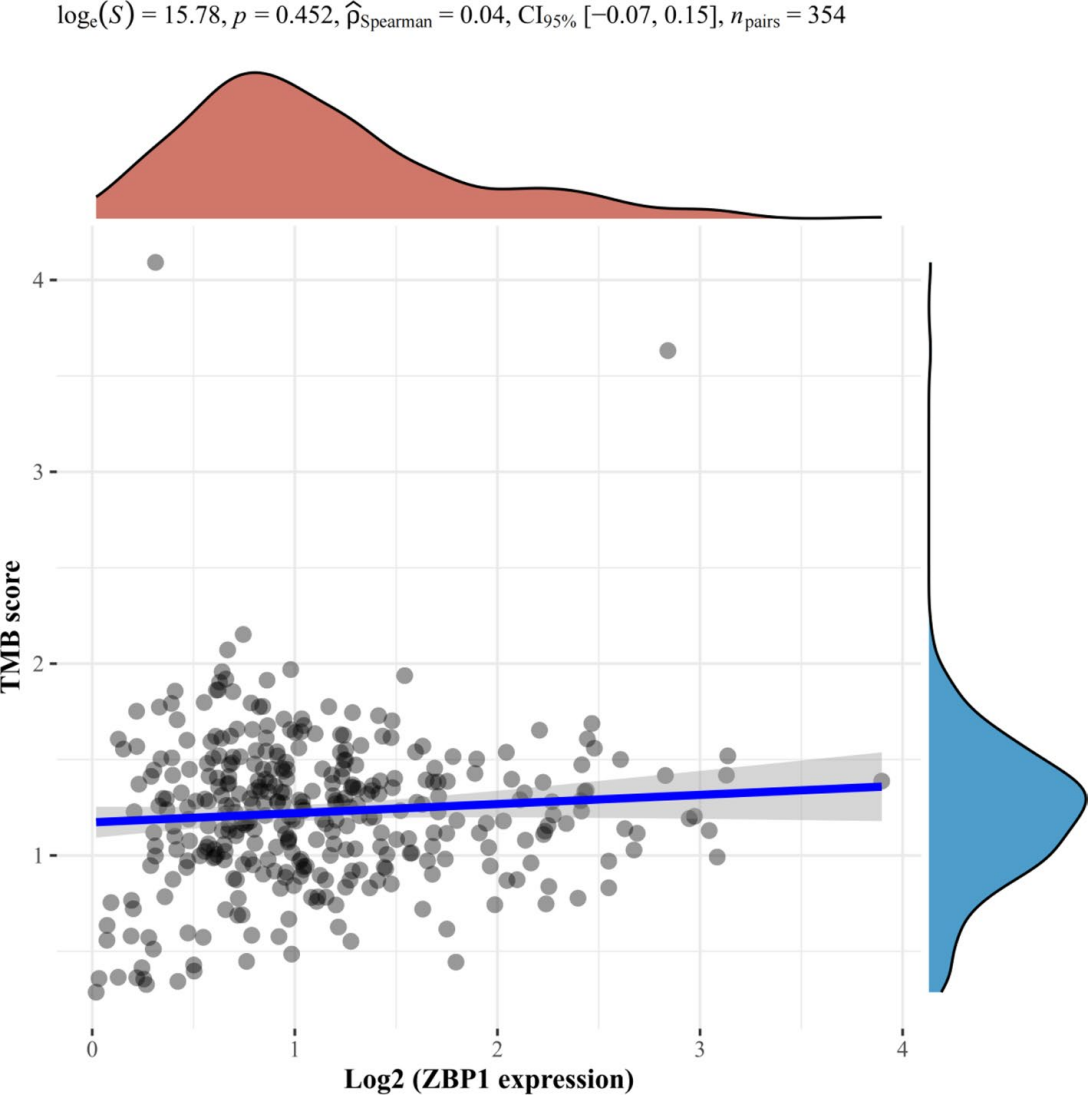
Spearman result of TMB and ZBP1 gene expression. The horizontal axis in the figure represents the expression distribution of genes, the vertical axis represents the distribution of TMB scores, and the density curve on the right represents the distribution trend of TMB scores; The upper density curve shows the distribution trend of gene expression; The top numerical value represents the correlation p-value, correlation coefficient and correlation calculation method

To further investigate the relationship between Tumor Mutation Burden (TMB) and necroptosis**-**related DEGs, Spearman correlation analysis of TMB and MYCN, CDKN2A and ZBP1 (Figs. 15, 16, 17) gene expression was made. The results revealed that TMB score was significantly correlated with expression of MYCN (p = 0.034) and CDKN2A (p < 0.05) instead of ZBP1 (p = 0.452).

### Assessment the OCLR scores of necroptosis related DEGs in KIRC

**Fig. 18:**
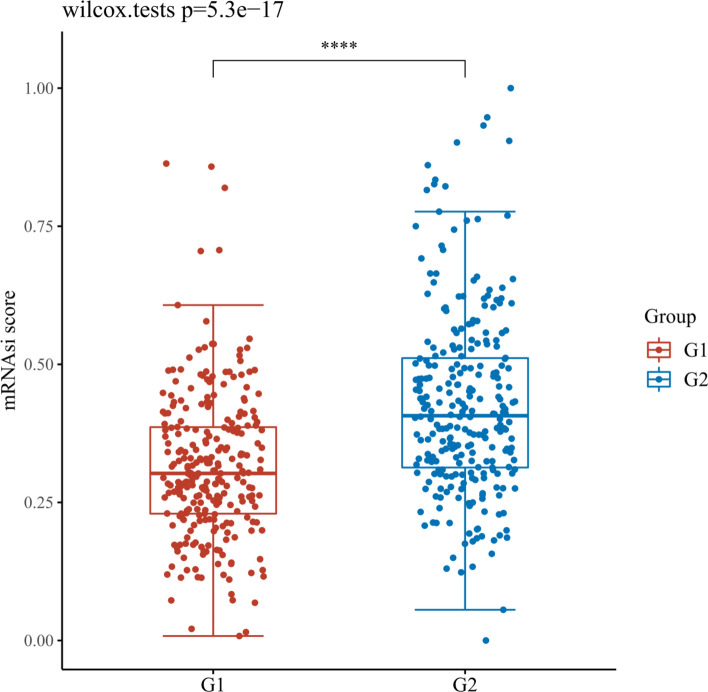
Scatter diagram reflecting the association of MYCN and OCLR score in KIRC. G1 is a high expression group and G2 is contrary. *p < 0.05, **p < 0.01, ***p < 0.001. The coordinates represent different groups of samples, and the vertical axis represents the distribution of dryness scores. Different colors represent different groups, and the upper left corner represents the significance p-value test method. The significance of two groups of samples was tested by wilcox test

**Fig. 19:**
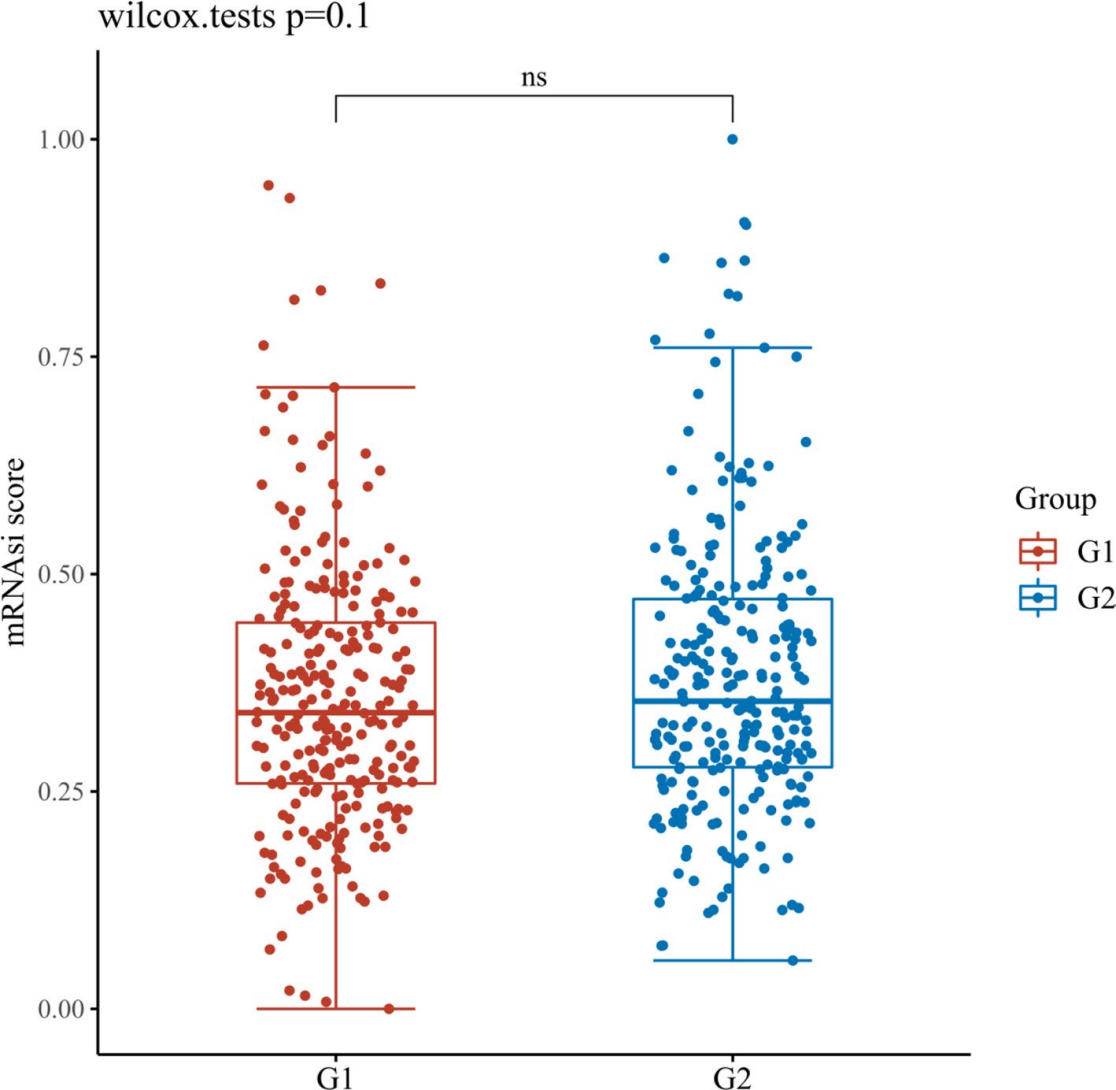
Scatter diagram reflecting the association of CDKN2A and OCLR score in KIRC. G1 is a high expression group and G2 is contrary. *p < 0.05, **p < 0.01, ***p < 0.001. The coordinates represent different groups of samples, and the vertical axis represents the distribution of dryness scores. Different colors represent different groups, and the upper left corner represents the significance p-value test method. The significance of two groups of samples was tested by wilcox test

**Fig. 20:**
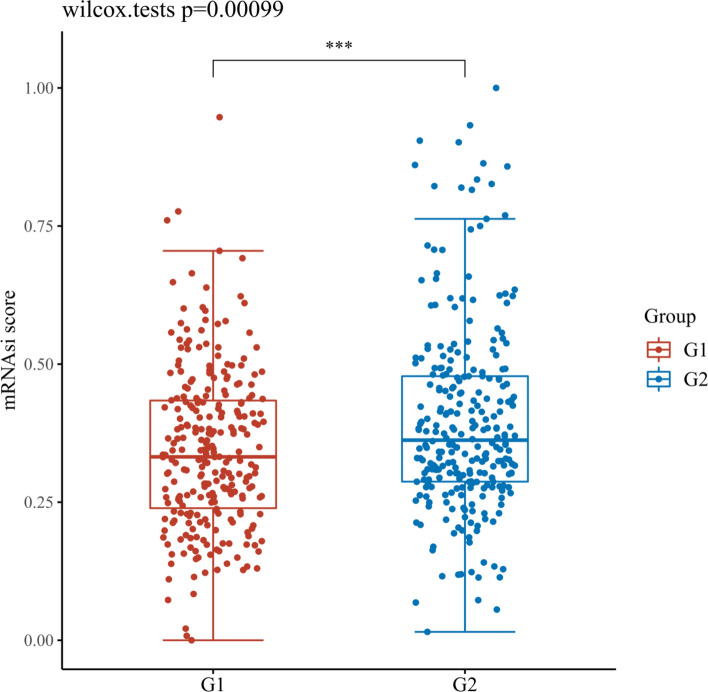
Scatter diagram reflecting the association of ZBP1 and OCLR score in KIRC. G1 is a high expression group and G2 is contrary. *p < 0.05, **p < 0.01, ***p < 0.001. The coordinates represent different groups of samples, and the vertical axis represents the distribution of dryness scores. Different colors represent different groups, and the upper left corner represents the significance p-value test method. The significance of two groups of samples was tested by wilcox test

After OCLR scoring, the results showed that except CDKN2A, the content of MYCN and ZBP1 in tissues was significantly different from KIRC dryness, as shown in Figs. 18, 19, 20. It is speculated that MYCN and ZBP1 will affect the similarity between KIRC cells and stem cells in different degrees, which means that they will affect the progress of tumor.

### Validation of the expression of DEGs in clinical tissue samples

**Fig. 21:**
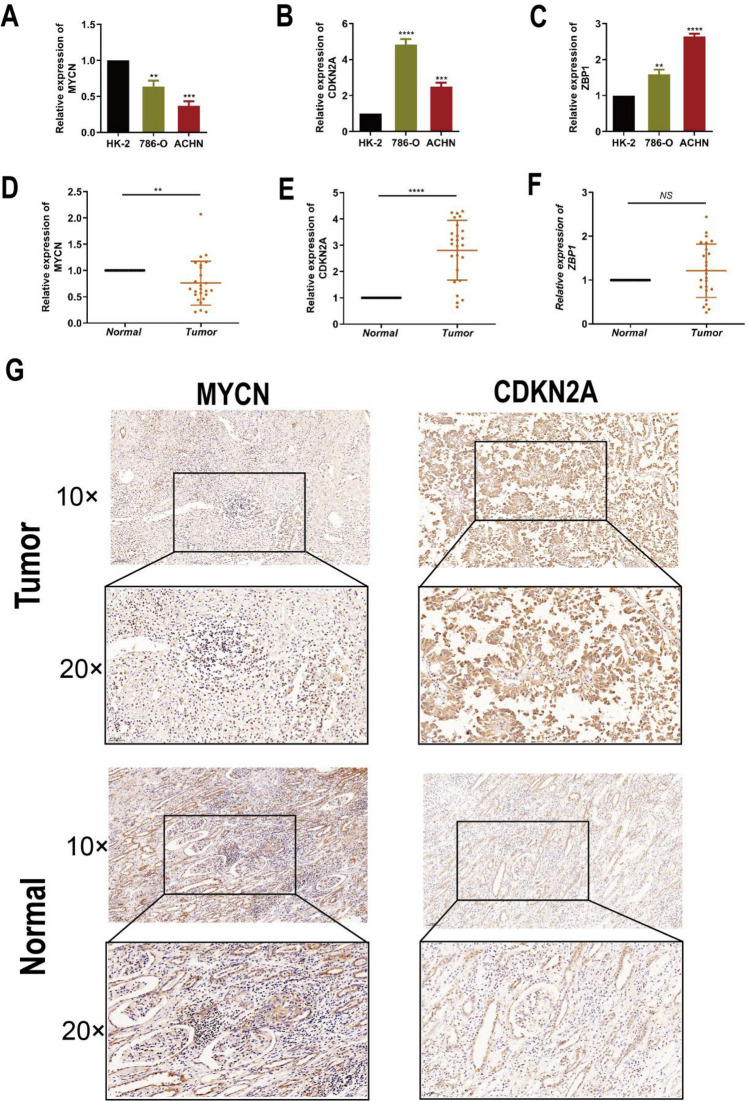
The expression of these genes in KIRC sample, adjacent normal tissues and cell lines. **A**–**C** q-RTPCR result of MYCN **A**, CDKN2A **B** and ZBP1 **C** in KIRC cell lines. **D**–**F** q-RTPCR analysis of MYCN **A**, CDKN2A **B** and ZBP1 **C** in paired KIRC tissues (n = 25). **G** Representative images of MYCN and CDKN2A protein in KIRC tissues and nearby normal tissues. Magnification:× 5, × 20. $$*\mathrm{p}<0.05, **\mathrm{p}<0.01, ***\mathrm{p}<0.001, ****\mathrm{p}<0.0001$$

In order to determine the actual content of three genes in KIRC, researchers implemented qRT-PCR in KIRC cell samples. The specific content of the four genes in the normal kidney cell line (HK-2 cell) was obtained. According to the comparative analysis between groups, the content of CDKN2A in KIRC cells is more than that in normal kidney cells. On the contrary, the content of MYCN in KIRC cells is less, as shown in Fig. 21A–C. Further analysis showed that the expression levels of MYCN and CDKN2A were basically the same in the pathological tissues and adjacent normal kidney tissues, and there was no significant difference in ZBP1 (Fig. 21D–F). IHC method is also introduced to determine the actual content of MYCN and CDKN2A protein in tissues. We found that compared with normal kidney tissue, the content of the above two indicators in the pathological tissue is more (Fig. 21G). The quantitative diagram of IHC is shown in Tables 1, 2.

**Table 1:** Expression of MYCN in 25 patients with RCC and adjacent tissues

Group	High expression	Low expression	High expression
			rate (%)
Tumor tissue	11	14	44.0
Adjacent tissue	19	6	76.0

**Table 2:** Expression of CDKN2A in 25 patients with RCC and adjacent tissues

Group	High	Low	High expression
	expression	expression	rate (%)
Tumor tissue	21	4	84.0
Adjacent tissue	7	18	28.0

### CDKN2A promotes and MYCN inhibits migration of KIRC cells in vitro

**Fig. 22:**
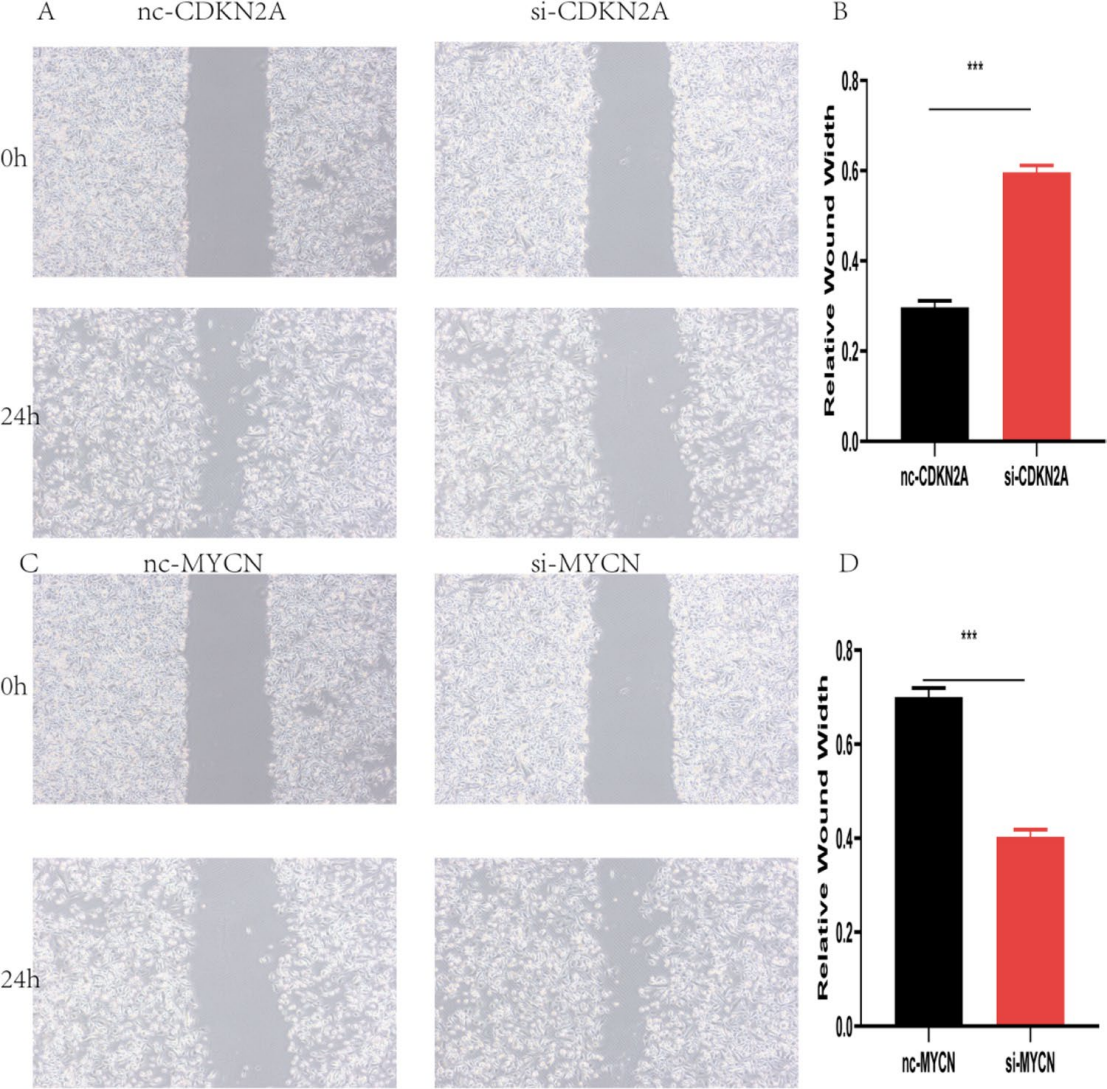
CDKN2A promotes and MYCN inhibits migration in ACHN cell line. **A** siRNA interference experimental group (si-CDKN2A) and control group (nc-CDKN2A) under wound healing assay. **B** Quantized histogram of **A**. **C** siRNA interference experimental group (si-MYCN) and control group (nc-MYCN) under wound healing assay. **D** Quantized histogram of C$$*\mathrm{p}<0.05, **\mathrm{p}<0.01, ***\mathrm{p}<0.001$$

After interfering with the expression of CDKN2A and MYCN, the results of wound healing assay showed that compared with the control group, the cell migration ability of the si-CDKN2A group was significantly reduced (P < 0.05) and the cell migration ability of the si-MYCN group was significantly induced (Fig. 22A–D).

### Validation of the necroptosis-related-DEGs

**Fig. 23:**
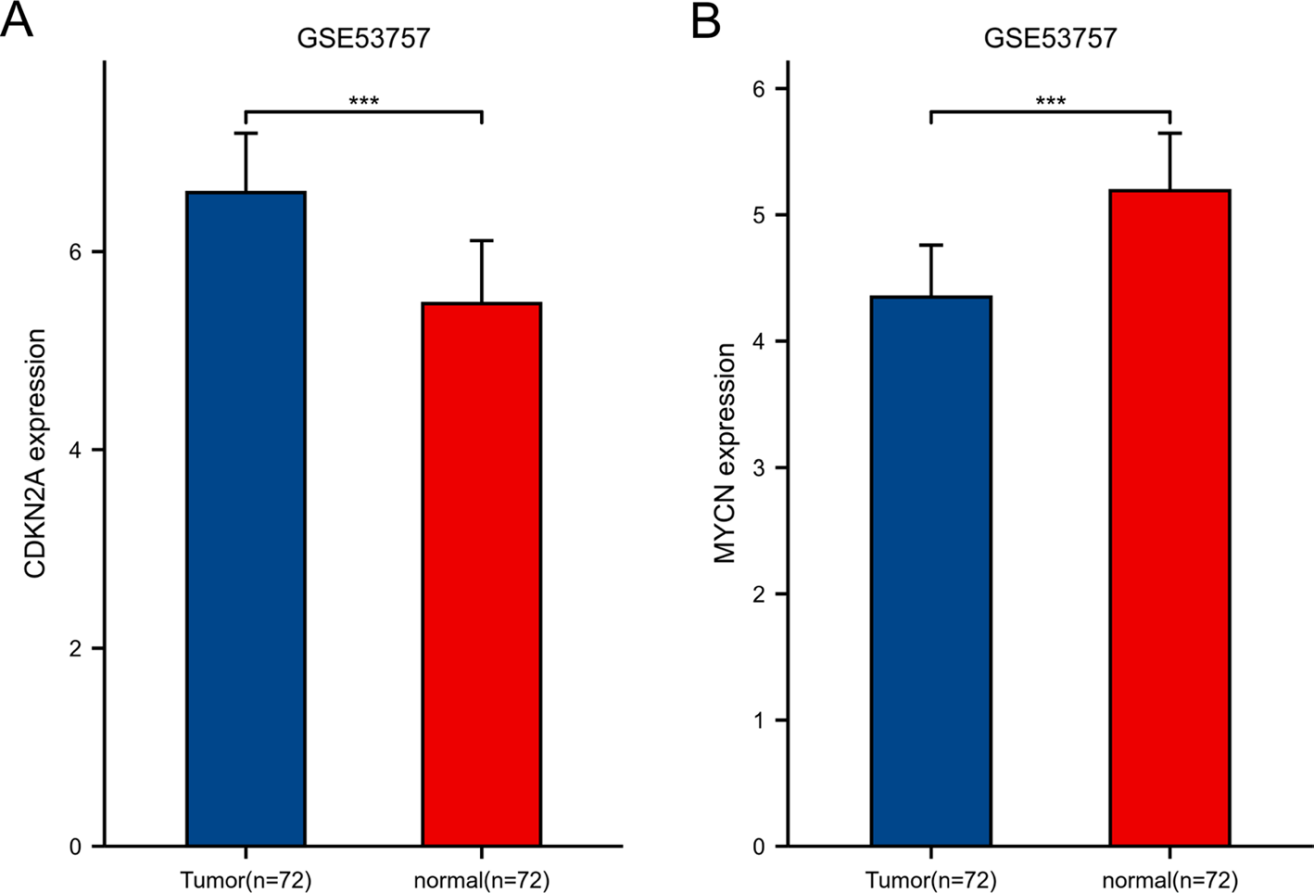
Differential expressions of CDKN2A **A** and MYCN **B** validated by GSE53757 datasets.$$*\mathrm{p}<0.05, **\mathrm{p}<0.01, ***\mathrm{p}<0.001.$$ The horizontal axis represents different groups of samples, the vertical axis represents the distribution of gene expression, and different colors represent different groups. The significance of two groups of samples was tested by wilcox test

**Fig. 24:**
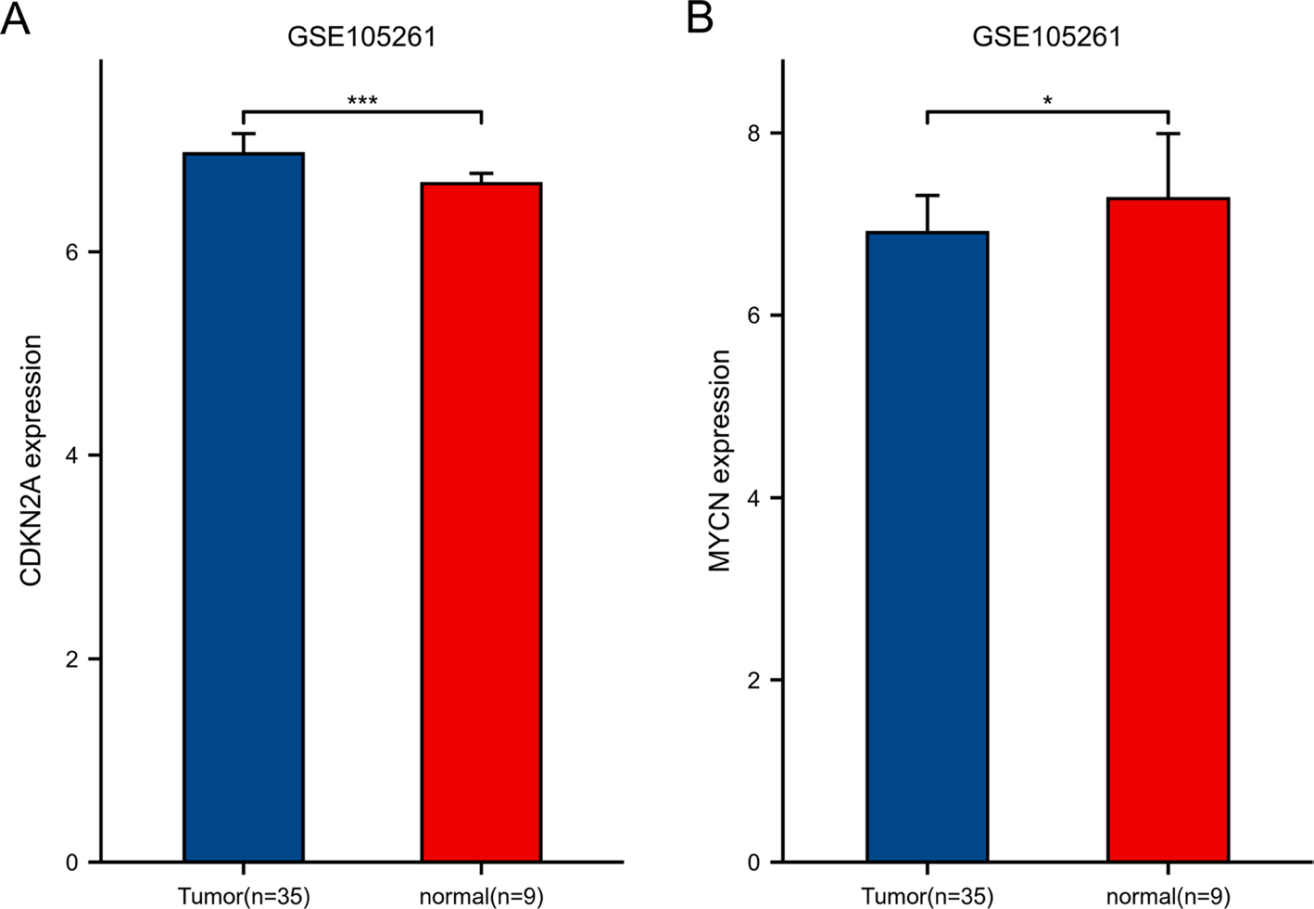
Differential expressions of CDKN2A **A** and MYCN **B** validated by GSE105261 datasets.$$*\mathrm{p}<0.05, **\mathrm{p}<0.01, ***\mathrm{p}<0.001.$$ The horizontal axis represents different groups of samples, the vertical axis represents the distribution of gene expression, and different colors represent different groups. The significance of two groups of samples was tested by wilcox test

In GSE53757 and GSE105261 dataset, the expressions of CDKN2A gene was significantly higher in KIRC tissues than in normal ones; According to the comparative analysis between groups, the content of MYCN gene in KIRC is higher in the two groups, and the difference is significant (Figs. 23, 24). In this paper, the clinical characteristics of patients were statistically analyzed. See Table 3 for details; The pathological characteristics are summarized. See Table 4 for details. It can be seen that the amount of MYCN content in tissues is related to t phase, m phase and OS. Further analysis shows that the amount of CDKN2A content is also related to t phase, m phase and OS.

**Table 3:** The association of the MYCN level and different variables and overall survival

Characteristic	Low expression of MYCN	High expression of MYCN	p
n	13	12	
T stage, n (%)			0.021
T1-2	7 (53.8%)	11 (91.7%)	
T3-4	6 (46.2%)	1 (8.3%)	
N stage, n (%)			0.124
N0	10 (76.9%)	6 (50.0%)	
N1	3 (23.1%)	6 (50.0%)	
M stage, n (%)			0.048
M0	7 (53.8%)	11 (91.7%)	
M1	6 (46.2%)	1 (8.3%)	
OS event, n (%)			< 0.05
Alive	4 (30.8%)	7 (58.3)	
Dead	9 (69.2%)	5 (41.7%)

**Table 4:** The association between the CDKN2A level and different pathological variables and overall survival in our center

Characteristic	Low expression of CDKN2A	High expression of CDKN2A	p
n	10	15	
T stage, n (%)			0.015
T1-2	7 (70.0%)	5 (33.3%)	
T3-4	3 (30.0%)	10 (66.7%)	
N stage, n (%)			0.234
N0	6 (60.0%)	7 (46.70%)	
N1	4 (40.0%)	8 (53.3%)	
M stage, n (%)			0.039
M0	7 (70.0%)	6 (40.0%)	
M1	3 (30.0%)	9 (60.0%)	
OS event, n (%)			< 0.05
Alive	8 (80.0%)	4 (26.7%)	
Dead	2 (20.0%)	11 (63.3%)	

## Discussion

Renal cell carcinoma (RCC) is one of the malignant tumors with high incidence rate of urinary system, which has serious physical harm. The incidence rate of the world is increasing. About 403 thousand of new cases occur annually, 175 thousand of deaths, about 4% of malignant diseases in adults are renal cell carcinoma [28], which ranks the first [29] in the annual mortality rate of urinary tumors. Many renal cell carcinomas still have no obvious symptoms in the advanced stage. Only about 20% of the patients with the three classic symptoms (hematuria, abdominal mass and pain). 50% of patients accidentally found through imaging examination during physical examination that about 16% of renal cell carcinoma had metastasized at the time of diagnosis. Most patients can only use palliative treatment, with poor prognosis and 5 year survival rate less than 10% [30]. Therefore, actively looking for sensitive markers of renal cell carcinoma plays an important role in the diagnosis of renal cell carcinoma, which has always been the focus of renal cell carcinoma research. At present, the prognosis of KIRC is poor, the disease is difficult to be effectively controlled in a short time, and the probability of tumor recurrence is high. From the current situation, the medical community has not yet identified the sensitive biomarkers of KIRC. The research team hopes to find effective targeted drugs through the mechanism of KIRC, in order to improve the quality of life of patients.

Relevant research results show that necrosis can inhibit cancer to a certain extent, but it may also aggravate the disease. Different tumor types play different roles in necrosis. In addition, at different stages of disease progression, necrosis also has two sides. For example, due to the low content of RIPK3, necrosis cannot play an inhibitory role, so as to promote tumor growth [31]. In human tumor samples, researchers found that the content of RIPK3 was reduced, such as acute myeloid leukemia [32], chronic lymphocytic leukemia [33], as well as common colorectal cancer [34] and breast cancer in clinical practice. Relevant research results show that low RIPK3 content will directly reduce the survival rate of ovarian cancer [35], colorectal cancer and breast cancer. In addition, many key mediating necrotic apoptosis molecules are downregulated in cancer, such as ubiquitination enzyme CYLD in chronic lymphocytic leukemia (CLL). CYLD is a deubiquitinase that promotes necroptosis and plays an important role in the process of necroptosis.

Other studies have shown that necrosis can also make the tumor expand continuously through various ways, and then aggravate the disease. Tumor cell metastasis is the main cause of cancer patients' death. Metastasis refers to individual tumor cells settling in other distant organs through the circulatory system and continuing to grow. Recent studies have shown that the extravasation of tumor cells can accelerate the metastasis and diffusion of diseased cells. These pathological cells further activate death receptor 6 to accelerate endothelial cell necrosis. So as to reduce tumor cell extravasation and metastasis. Therefore, blocking necroptosis of endothelial cells may be a potential clinical treatment to inhibit tumor cell metastasis [36]. The mechanism of metastasis is complex and closely related to tumor-related microenvironment. Previous research results show that in pancreatic ductal adenocarcinoma, if RIPK3 is knocked out, the diseased cells will necrosis. In this process, it will release a large number of soluble cytokines, which will further bind to receptors on inflammatory cells, such as SAP130 and its homologous receptor mincle, so as to trigger the immunosuppressive tumor microenvironment and promote the progress of pancreatic ductal adenocarcinoma [37]. In general, these studies show that tumor necroptosis occurs in vivo, It can promote tumor by inducing tumor-promoting immune microenvironment.

The use of necrosis to intervene in tumor provides a new idea for cancer treatment, but its safety and effectiveness need to be further verified. Some scholars pointed out that necrosis inhibits the migration of diseased cells through different signal pathways, so this method is worth promoting. Recent studies have also verified this view. However, some scholars are skeptical. They found that many cancer cells have defects in the necrosis mechanism, which may affect healthy cells and cannot ensure their safety. In fact, in addition to the existing evidence that natural products such as shikonin can induce necroptosis, many traditional chemotherapy or molecular targeted drugs that have recently been approved for clinical trials have been identified as cancer necrosis inducers in some cancer types [38, 39], such as VEGFR inhibitors, m-TOR inhibitors, etc. Inducing necrosis in cancer cells is not necessarily toxic to normal cells, and even leads to serious side effects in vivo. At the same time, in order to enhance the specificity of drugs, necrosis inducers can also be considered to be combined with tumor targeted drugs to increase the specificity of drugs on tumor cells [40], such as IFN- γ Combined with proteasome inhibitor bortezomib to inhibit tumor cells. In the past, people have not conducted a large number of studies on the correlation between KIRC and the prognosis of necrosis. This paper fills up the gap in this field. At present, surgery, chemotherapy and radiotherapy are the most widely used KIRC treatment methods in clinical practice. Among these therapies, surgical resection has the advantages of quick effect and significant effect, but it is not very suitable for patients with advanced diseases. In this case, people put forward cancer immunotherapy.

This paper has made a comprehensive analysis of the genes in GSE168845, 67 necroptosis-related genes to obtain 3 co-expressed necroptosis related DEGs. The results of this study showed that compared with normal tissues, the expression of CDKN2A and ZBP1 in cancer cells was significantly increased, while the expression of MYCN was on the contrary. It can be concluded that both would affect the pathological changes of tumor. Kaplan–Meier analysis showed that the expression levels of CDKN2A and ZBP1 were negatively correlated with the prognosis of patients, while the expression of MYCN was positively correlated with the prognosis. Comparative analysis showed that this result was consistent with the results of the DEGs prognosis model. In order to further study the relationship between these three kinds of DEG and tumor, this paper also studied the relationship between their expression level and tumor stage, tissue morphology, patient age and other factors during the study. The results of calibration curve and nomogram showed that these three genes showed good prognostic value. Its expression level was significantly correlated with immune infiltration.

MYCN is a small molecular protein that plays an important regulatory role in cell physiological activities [41]. Its gene was discovered in 1983 [42]. MYCN has multiple regulatory effects on cells, such as promoting cell proliferation and apoptosis [43, 44]. Relevant experimental studies show that MYCN has a certain regulatory effect on the expression of pro-apoptotic regulatory factor NOXA. The study found that under the induction of MYCN, the sensitivity of cells to toxic drugs was significantly improved. The inactivation of MYCN will promote the apoptosis of cancer cells, thus inhibiting the development of tumor [45, 46]. Aging and/or apoptosis are mainly related to their regulation of adaptive immune response elements (PDL1, CD47), and there is a positive correlation between MYCN and the level of these elements. CDKN2A is a typical tumor suppressor gene, which can regulate the physiological activities of cancer cells in many ways. It is widely expressed in human cells. It can encode p16 (p16INK4a) and p14ARF proteins, both of which can regulate cell cycle and thus inhibit tumor. CDKN2A mutation is very common in tumors. Its mutations mainly include homozygous deletion, mutation and promoter abnormal methylation of p16INK4a / p14ARF gene [47]. ZBP1 was originally found as an up-regulated gene in the transcriptome study of tumor cells by interferon and named DLM-1. Later, it was found that it has the ability to bind to left-handed DNA, that is, Z-DNA, so it is officially called ZBP1. ZBP1 protein has two Z-DNA binding domains at the N-end and two Rhim domains in the middle, so it can bind to RIPK1/3. Recently, relevant studies found that ZBP1 is associated with the recognition of virus after influenza A virus (IAV) infection and the regulation of the death mode of virus infected cells (including apoptosis, programmed necrosis and cell scorch death). In 2020, Wang et al. [48] found that histone methyltransferase SETDB1 was involved in the disease process of inflammatory bowel diseases (IBD). The specific mechanism is that the reduced expression of SETDB1 gene will lead to the activation and transcription of endogenous retrovirus. A large number of viral RNA in cells will activate ZBP1 protein, and then ZBP1 protein will recruit RIPK3 to promote programmed cell necrosis.

There are three studies reporting necroptosis-related genes in KIRC [49–51], however, these three articles only roughly described the necroptosis-related genes level in RCC. We enriched these necroptosis-related genes and found that these necroptosis-related genes may be related to immunity in renal cell carcinoma. We also determined the relation between these genes and stemness, TMB, ICB responses, immune checkpoint in renal cell carcinoma. CDKN2A can accelerate the invasion and migration of renal cancer cells and MYCN inhibits the invasion and migration of renal cancer cells through wound healing assay and transwell invasion assays.

There are also some limitations in this study. We only conducted preliminary expression research on these three necroptosis-related genes. The functional analysis needs to be deeply conducted in future.

## Conclusion

In this study, necroptosis associated genes with high prognostic value were obtained based on bioinformatics method, and the prognostic risk model was established. A strong correlation was determined between necroptosis-related genes and immune score, ICP and OCLR score. MYCN and CDKN2A can be regarded as promising targets for immunotherapy linked with necroptosis in KIRC.

## Data Availability

Publicly available datasets were analyzed in this study. This data can be found here: https://tcga.xenahubs.net.
